# Association between a selective 5-HT_4_ receptor agonist and incidence of major depressive disorder: an emulated target trial

**DOI:** 10.1192/bjp.2024.97

**Published:** 2024-09-01

**Authors:** Angharad N. de Cates, Catherine J. Harmer, Paul J. Harrison, Philip J. Cowen, Anton Emmanuel, Simon Travis, Susannah E. Murphy, Maxime Taquet

**Affiliations:** 1 Department of Psychiatry, University of Oxford, Warneford Hospital, Oxford, UK; 2 Institute for Mental Health, University of Birmingham, Birmingham, UK; 3 Oxford Health NHS Foundation Trust, Warneford Hospital, Oxford, UK; 4 Oxford Centre for Human Brain Activity and Oxford Centre for Functional MRI of the Brain, Wellcome Centre for Integrative Neuroimaging, Department of Psychiatry, University of Oxford, Oxford, UK; 5 GI Physiology Unit, UCLH, London, UK; 6 Kennedy Institute and Translational Gastroenterology Unit, University of Oxford, UK

## Abstract

**Background:**

The serotonin 4 receptor (5-HT_4_R) is a promising target for the treatment of depression. Highly-selective 5-HT_4_R agonists, such as prucalopride, have antidepressant-like and pro-cognitive effects in pre-clinical models, but their clinical effects are not yet established.

**Aims:**

To determine whether prucalopride (a 5-HT_4_R agonist and licensed treatment for constipation) is associated with reduced incidence of depression in individuals with no past history of mental illness, compared to anti-constipation agents with no effect on the central nervous system.

**Method:**

Using anonymised routinely collected data from a large-scale US electronic health records network, we conducted an emulated target trial comparing depression incidence over one year in individuals without prior diagnoses of major mental illness who initiated treatment with prucalopride versus two alternative anti-constipation agents which act by different mechanisms (linaclotide and lubiprostone). Cohorts were matched for 121 covariates capturing sociodemographic factors, and historical and/or concurrent comorbidities and medications. The primary outcome was a first diagnosis of major depressive disorder (ICD-10 code F32) within one year of the index date. Robustness of the results to changes in model and population specification was tested. Secondary outcomes included a first diagnosis of six other neuropsychiatric disorders.

**Results:**

Treatment with prucalopride was associated with significantly lower incidence of depression in the following year compared to linaclotide (HR 0.87, 95% CI 0.76-0.99, *p*=0.038, n=8572 in each matched cohort) and lubiprostone (HR 0.79, 95% CI 0.69-0.91, *p*<0.001, n=8281). Significantly lower risks of all mood disorders and psychosis were also observed. Results were similar across robustness analyses.

**Conclusions:**

These findings support pre-clinical data and suggest a role for 5-HT_4_R agonists as novel agents in the prevention of major depression. These findings should stimulate randomised controlled trials to confirm if these agents can serve as a novel class of antidepressant within a clinical setting.

## Introduction

Depression is a leading cause of disability worldwide and improving its treatment is a global health priority. (1) One third of patients do not achieve remission with current treatment approaches, which has a significant impact on occupational and social functioning. (2) First-line antidepressants also have limited efficacy in treating some symptom clusters such as cognitive impairment. (3) Within this context, there is a pressing need for novel antidepressant targets. (4) However, drug discovery in psychiatry is notoriously challenging, with fewer than half the 5-year new FDA approvals in neurology. (5) One promising strategy is the repurposing of drugs known to act on receptors in the central nervous system and used to treat conditions affecting other organ systems. (6)

**Figure 1:**
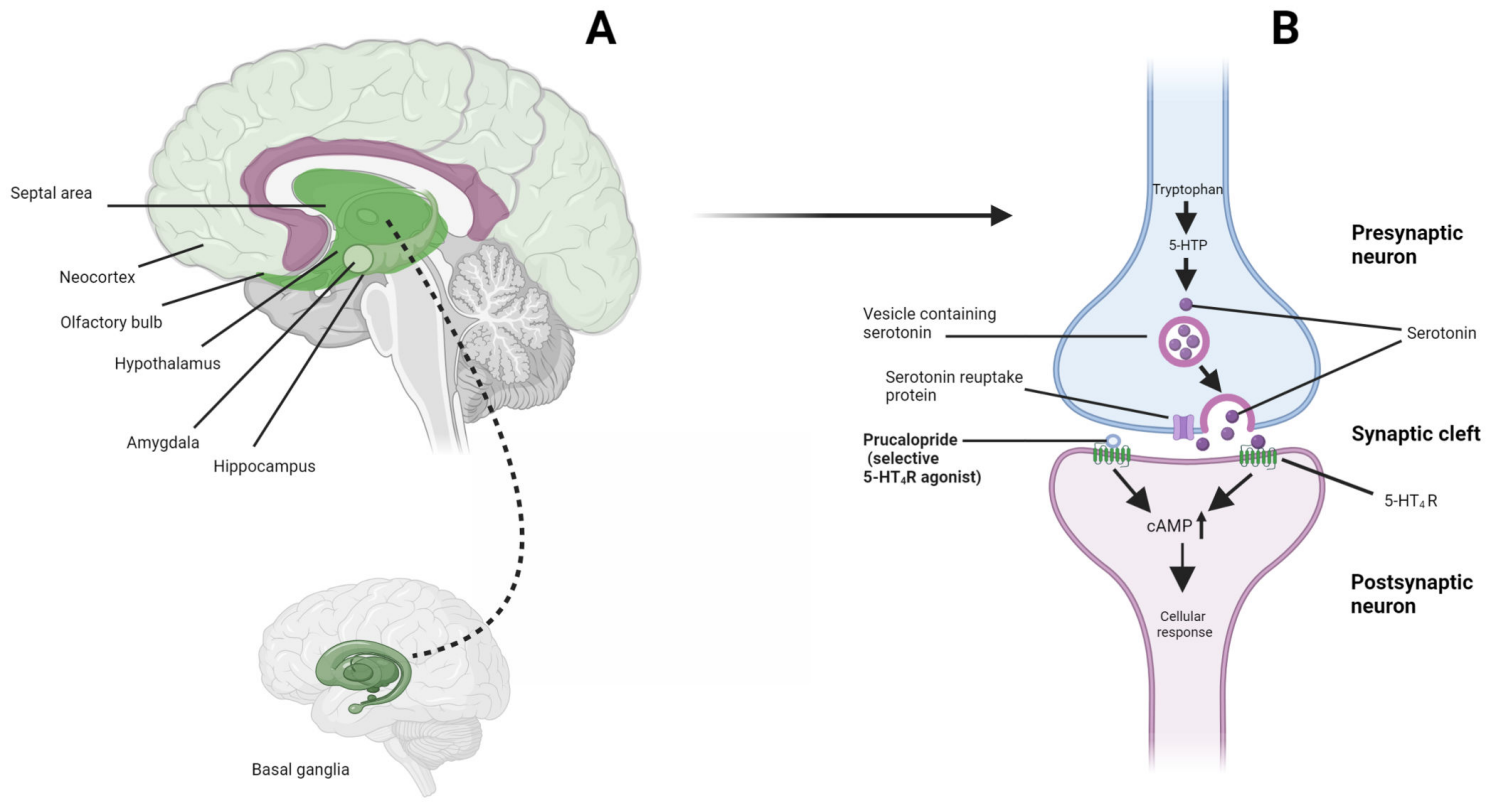
Locations of 5-HT_4_ receptors in the brain and actions of prucalopride (A) Brain regions where 5-HT_4_ receptors are particularly highly expressed (see Beliveau et al 2017, J Neurosci, 37(1):120-128). Darker green indicates regions of highest expression (i.e. basal ganglia); lighter green indicates relatively lower expression (i.e. neocortex); (B) Action of prucalopride at a serotonin synapse as a highly-selective agonist at transmembrane G protein-coupled 5-HT_4_ receptors. cAMP = cyclic AMP, 5-HTP = 5-Hydroxytryptophan, 5-HT_4_R = Serotonin 4 receptor.

A first step in drug repurposing is the identification of a candidate compound. Serotonin 4 postsynaptic receptors (5-HT_4_R) are widely expressed in the brain, particularly in regions and networks related to mood and cognitive functioning (see Figure 1). (7) 5-HT_4_R agonists have rapid antidepressant-like effects in animal models of depression (8, 9) and a facilitatory effect on rodent behavioural tasks of learning and memory. (10) In humans, there is also emerging experimental evidence to support a role for the 5-HT_4_ receptor in depression and cognition. (11, 12) Brain 5-HT_4_R binding is reduced in depressed patients compared with healthy controls, and associated with deficits in memory performance. (11)

However, clinical evidence for the effect of 5-HT_4_R agonists on depression is lacking. Emulated target trials allow assessment of the effect of a treatment on a clinical outcome outside the existing license using observational data. (13) Using this approach, we assessed whether exposure to the 5-HT_4_R agonist prucalopride (an anti-constipation agent acting on 5-HT_4_R in the gut, but also with good brain penetration (14)) is associated with a lower risk of depression within one year since first prescription compared to two alternative anti-constipation agents which have no known effect on 5-HT_4_Rs or the central nervous system. We hypothesised that participants on prucalopride would show a lower risk of depression within one year.

## Method

### Study design and data collection

The TrinetX US Collaborative Network was used for this study: a network of anonymised electronic health records data from 60 healthcare organisations in the United States (US). (15) This network contains anonymised data from over 100 million patients, including demographics, diagnoses as ICD-10 codes, and medications (see Supplementary Methods 1 for details). In brief, this network allows access to over 100 million patients with data including demographics, diagnoses and ICD-10 codes, medications, procedures, measurements (e.g. blood pressure, body mass index) and health care visits. Data is included from both primary and specialist healthcare organisations, and involves both patients insured and not insured under the standard US system. Each healthcare organisation remains anonymous within TriNetX, and is able to provide data with the necessary consents and approvals as long as research is the sole use. The process of data de-identification is attested by a qualified expert as defined in Section 164.514(b)(1) of the HIPPA Privacy Rule. (16) No further ethical approval is needed. As we used anonymised routinely collected data, no participant consent was required. We followed the approach of Hernán and Robins to emulate a target trial using electronic health records from TriNetX. (17) The different components of the target and emulated trials are summarised below and detailed in Supplementary Methods and Supplementary Table 1. We followed STROBE guidelines (see Supplement).

### Participants and exposure

The primary cohort included individuals with a first prescription of prucalopride before the start date of analysis (25^th^ January 2024). Two comparator cohorts were defined as individuals with a first prescription of (i) linaclotide (a guanylate cyclase 2C agonist) and (ii) lubiprostone (a chloride channel activator) before 25^th^ January 2024. For all cohorts, no age or sex restriction was applied but patients with pre-existing diagnoses of common and/or serious mental illness were excluded (ICD-10 codes for psychotic disorders [F20-F29], mood disorders [F30-F39], anxiety disorders [F40-F48], and mental disorders due to known physiological conditions including cognitive disorders [F01-F09]). Patients were censored at their last clinical encounter or when they died. An intention-to-treat analysis was emulated by including all individuals who received one prescription of the drug in the corresponding cohort. See Supplementary Methods 2 for details on cohort definition.

All three medications (prucalopride, linaclotide, and lubiprostone) are approved by the FDA for use in the US for chronic constipation, are third-line pharmacotherapy options (i.e. when laxatives alone are insufficient) in national guidelines, (18) and are available under Medicaid/Medicare.

### Covariates

Cohorts were matched using propensity score matching for 121 variables capturing sociodemographic factors, and concurrent or history of comorbitidies and medications that could be associated with differences in choices of anti-constipation treatment and/or with psychiatric disorders. These covariates were selected based on expert opinion as well as statistical differences between cohorts before matching (see Supplementary Methods 3 for details and Supplementary Table 2). Baseline characteristics of cohorts prior to matching (lifetime, 5 years and 1 year before inclusion) were checked (Supplementary Tables 3-5).

### Outcomes

The primary outcome was the incidence of a first diagnosis of major depressive disorder (F32) within one year of the index date. Secondary outcomes included incidence of a first diagnosis of six other common and/or serious neuropsychiatric disorders in the first year: (i) mood [affective] disorder (F30-F39), as an overarching outcome, as well as (ii) bipolar disorder (F31) specifically; (iii) anxiety disorder (F40-F48); (iv) dementia (any of F01-F03, G30, G31.0, G31.2, G31.83); (v) substance use disorder (F10-F19); (vi) psychotic disorder (F20-29).

### Secondary analyses

Details on all secondary analyses can be found in Supplementary Methods 5.

#### Robustness analyses

Robustness of the results to changes in outcome, cohort, and model specifications was tested under the following conditions (i) excluding from all cohorts inidviduals with a contraindication for any of the study medications; (ii) excluding those within the comparator (linaclotide / lubiprostone) cohort who have a prescription of prucalopride in the year prior to the index date, thus making cohorts mutually exclusive; (iii) excluding those from each cohort with a recorded prescription of the alternate drug in the one year following the index date; (iv) excluding those with additional pre-existing neuropsychiatric disorders; and (v) excluding patients with recent prescription of either of the two most common SSRI antidepressants (escitalopram and sertraline).

#### Negative control outcomes

To assess for potential unmeasured confounding, matched cohorts were compared in terms of a range of negative control outcomes that are not expected to be influenced by differences in anti-constipation medications. (19) Twenty negative control outcomes were selected, adapted from previous analyses using the TriNetX database, (20) and first occurrences analysed both individually and as a composite outcome. A full list of negative control outcomes is available in Supplementary Methods.

#### Additional comparisons

Influences of medication costs and Medicaid/Medicare availability were tested using additional comparisons (in terms of psychiatric and negative control outcomes):

- Linaclotide (similar price as prucalopride) vs. lubiprostone (cheaper than prucalopride)
- Plecanatide (same mechanism of action as linaclotide and available under Medicaid/Medicare at the same time as prucalopride) vs. linaclotide (available under Medicaid/Medicare earlier than prucalopride).

To assess whether differences in incidence of depression could be explained by differences in efficacy of anti-constipation drugs, we compared prucalopride to both comparator drugs in terms of incidence of an enema (taken to be an indicator of suboptimal anti-constipation treatment) over the one-year follow-up.

#### Interrupted time series analysis

We complemented the emulated target trial with an interrupted time series analysis comparing the trend in depression incidence in the 12 months after versus before the first prescription of prucalopride, and relative to the total number of people who had at least one health encounter within each month. We hypothesised a change in slope with a progressive reduction in the number of depression diagnoses after initiation of prucalopride. This analysis was also conducted for negative control outcomes.

### Statistical analysis

Propensity score 1:1 matching was achieved using a greedy nearest neighbour algorithm with caliper distance of 0.1. Matching for a covariate was considered adequate if the standardised mean difference between matched cohorts was less than 0.1. (15) Cumulative incidences over the one-year follow-up were estimated using the Kaplan-Meier estimator. The log-rank test was used to compare survival between matched cohorts and the Cox proportional hazard model was used to estimate hazard ratios (HR) with 95% confidence intervals (CI) (using the “survival” package in R 4.2.1). The proportional hazard assumption was tested with the generalised Schoenfeld approach. E-values were calculated for all comparisons in the primary analysis. (21) Statistical significance was set at two-sided *p*<0.05.

## Results

A total of 8694 patients had at least one prescription of prucalopride (mean [standard deviation, SD] age 48.8 [19.0], 75.8% female, 20.6% male), of which 8572 and 8281 were successfully matched 1:1 to patients with a first prescription of linaclotide or lubiprostone respectively (Table 1 for baseline characteristics of matched cohorts and Supplementary Tables 3-5 for baseline characteristics before matching).

**Figure 2:**
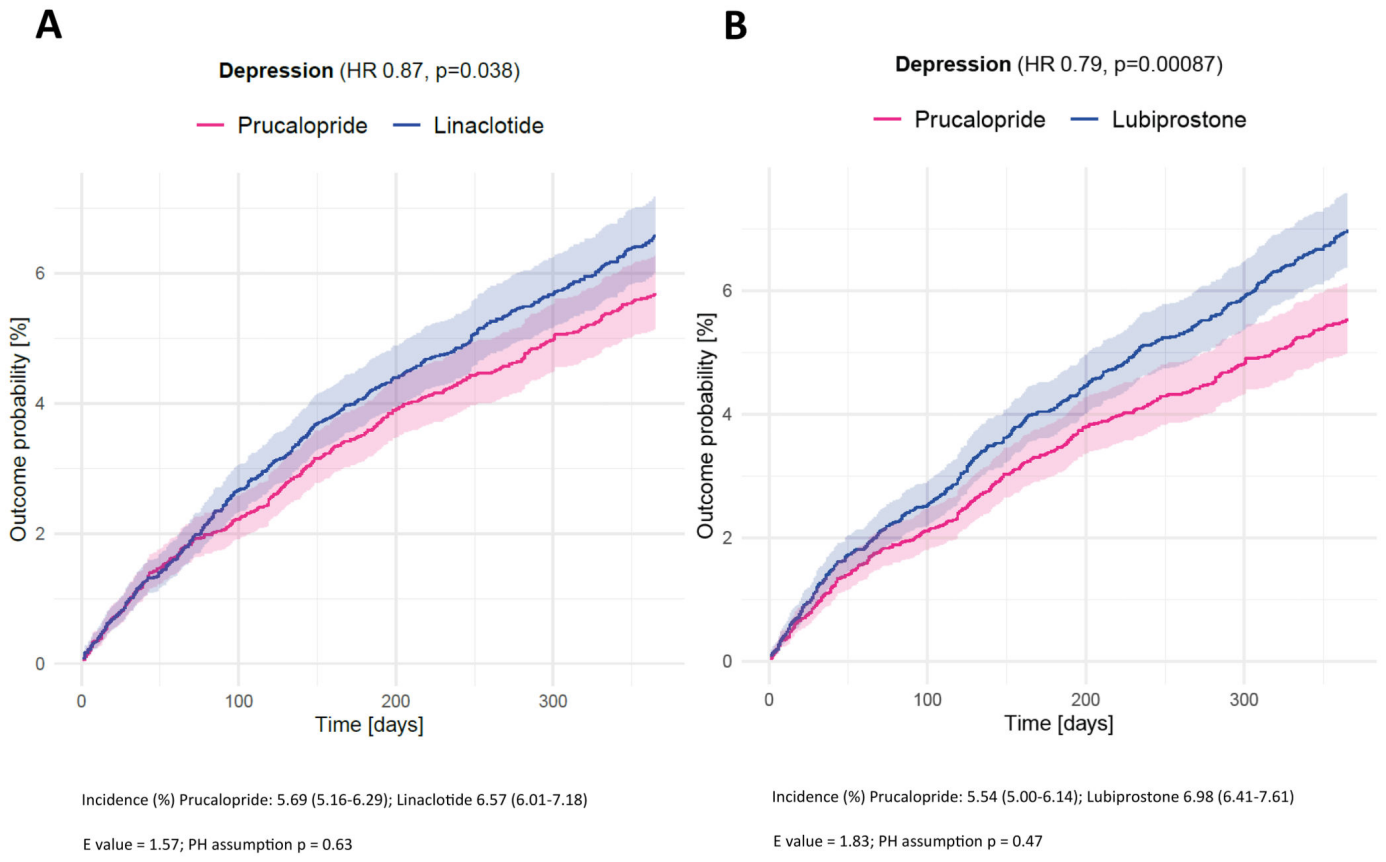
Kaplan-Meier curves showing the cumulative incidence of depression diagnosis over 12 months in those receiving prucalopride versus linaclotide (A) and prucalopride versus lubiprostone (B) The shaded areas around curves represent 95% confidence intervals. HR=Hazard ratio. PH=Proportional hazard.

In the one year following a first prescription, those prescribed prucalopride had a significantly lower incidence of depression (prucalopride versus linaclotide: HR 0.87, 95% CI 0.76-0.99, *p*=0.038, E-value 1.58; prucalopride versus lubiprostone: HR 0.79, 95% CI 0.69-0.91, *p*<0.001, E-value 1.83; Figure 2). There was no evidence of non-proportional hazards for either comparison (p=0.63 and 0.47 respectively).

These results were replicated in all robustness analyses for lubiprostone, and all but one for linaclotide (Supplementary Table 6). In particular, the findings held when we excluded participants who had a contraindication for any drug being compared, patients who crossed over to the other ‘arm’ of the comparison within the one-year follow-up, or patients who had a recent prescription of one of the two most common SSRI antidepressants. The only exception was the comparison with linaclotide when participants with a broader range of neuropsychiatric diagnoses were excluded, which resulted in a non-significant association of similar effect size (HR 0.87, 95% CI 0.76-1.00, *p*=0.058).

In terms of secondary outcomes (Table 2 and Supplementary Figures 1 and 2), compared with linaclotide and lubiprostone, those prescribed prucalopride had a lower incidence of all mood disorders (prucalopride versus linaclotide: HR 0.85 (95% CI 0.75-0.96), *p*=0.0074; versus lubiprostone HR 0.81 (95% CI 0.71-0.91), *p*<0.001) and psychotic disorder (prucalopride versus linaclotide: HR 0.27, 95% CI 0.11-0.67, *p*=0.0019; versus lubiprostone: HR 0.26, 95% CI 0.096-0.68, *p*=0.0023). Results for these two secondary outcomes were replicated in all robustness analyses (Supplementary Tables 7-11). Findings for other outcomes, including dementia, bipolar disorder, and substance misuse, varied in significance across analyses.

No significant differences in negative outcomes were observed (see Supplementary Tables 7-12). We also found no significant difference between matched cohorts in terms of risk of needing an enema (prucalopride versus linaclotide: HR 1.02, 95% CI 0.14-7.23, *p*=0.99; versus lubiprostone: HR 2.09, 95% CI 0.19-23.12, *p*=0.53).

There was no significant difference in risk of depression between linaclotide and lubiprostone (n=78,581 in each matched cohort, HR 1.00, 95% CI 0.95-1.04, p=0.83) indicating that medication price is unlikely to confound the association (Supplementary Table 13; for rationale see Methods). Similarly, there was no significant difference in incidence of depression between linaclotide and plecanatide (n=2405 patients in each matched cohort, HR 1.10, 95% CI 0.85-1.42, *p*=0.46), indicating that Medicaid/Medicare availability date is unlikely to confound the association (Supplementary Table 14).

**Figure 3:**
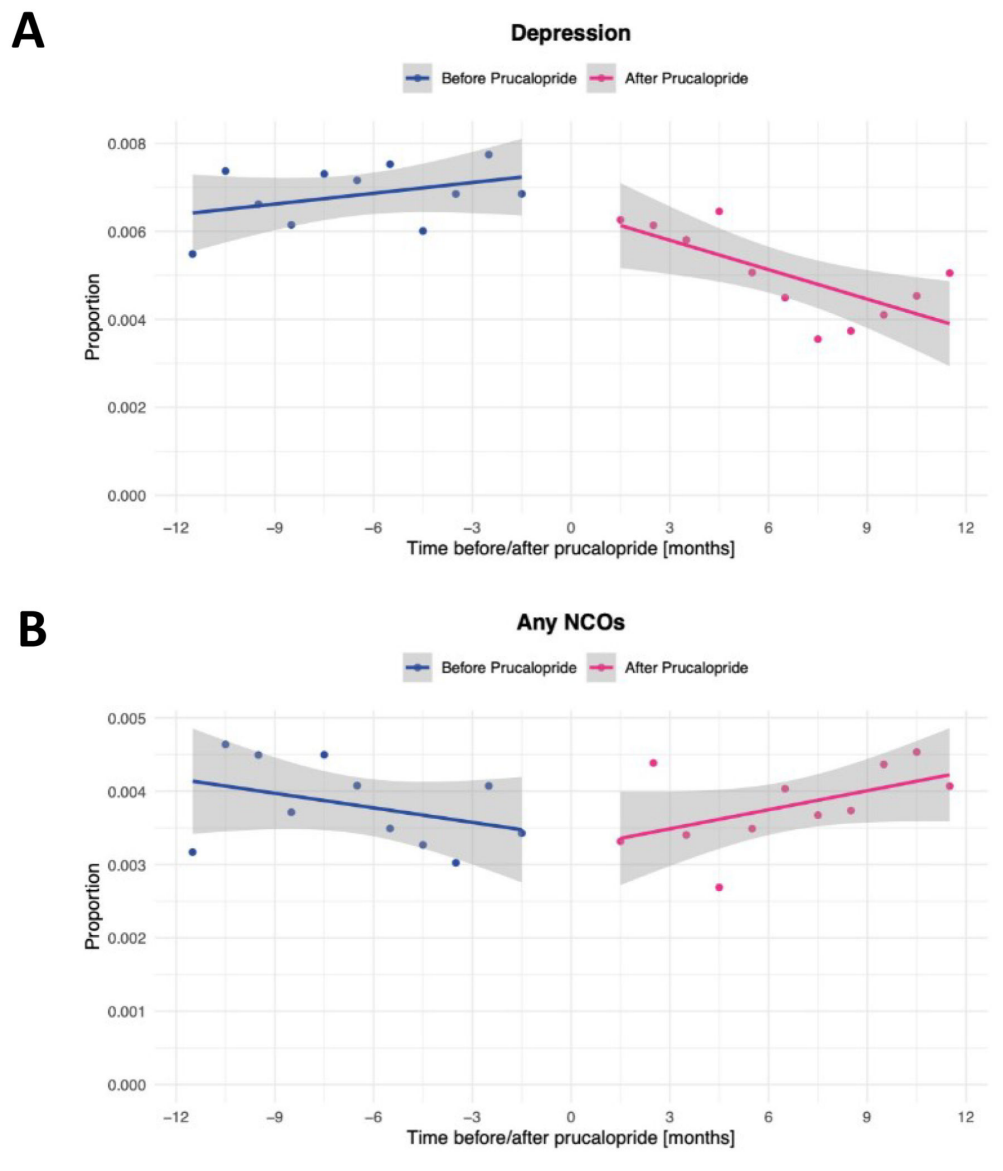
Interrupted time series analysis comparing the incidence of depression (A) and any negative control outcomes (B) before and after prucalopride prescription, shown as a proportion of people who had a healthcare encounter during each month.

In the interrupted time series analysis, the incidence of depression significantly decreased after prescription of prucalopride (-3.05 cases/10,000 people per month, 95% CI -4.95 – -1.14, p=0.0058; no evidence of auto-correlation, p=0.63; Figure 3(A)). This finding was robust when two months on either side of prucalopride prescription were included, and when absolute counts rather than incidence were analysed (Supplementary Table 15). Conversely, no decrease in incidence of negative control outcomes was observed over follow-up (+1.53 cases/10,000 people per month, 95% CI 0.12-2.93, p=0.047; Figure 3(B)).

## Discussion

In this emulated target trial, the highly-selective 5-HT_4_R agonist, prucalopride, was associated with a 13-21% lower risk of a first episode of depression within the following year compared to matched cohorts of patients prescribed drugs with similar indication but without action at the 5-HT_4_R (linaclotide and lubiprostone). This study is the first to consider the mental health effects of prucalopride using clinical health records and adds to a growing evidence base highlighting the potential role of 5-HT_4_R agonists in depression. It may also help to inform the choice of anti-constipation drug in people with chronic constipation who are at risk of depression

The research question of this study lends itself well to an emulated target trial for two reasons: (i) all three drugs are third-line interventions for chronic constipation (18) and the choice between the three is largely driven by clinician’s preference thus limiting indication and selection bias, and (ii) there is no *a priori* reason for patients and clinicians to have believed that these drugs would have differential effects on mental health thereby limiting detection bias.

There are several mechanisms through which 5-HT_4_R agonism might reduce depression risk. First, 5-HT_4_R agonists may have a direct effect on mood after penetrating the blood-brain barrier, with evidence of increased BDNF/CREB production and direct action on the raphe nucleus in pre-clinical studies. (8, 9) Second, 5-HT_4_R agonists have pro-cognitive effects in animal (10) and human models of learning. (12, 22) Third, the association between prucalopride and depression may be mediated by action on the gut-brain axis, including effects on the microbiome or gut serotonin. (23) Fourth, prucalopride may be acting to increase stress resilence: 5-HT_4_R agonists decrease stress-induced depressive-type behaviours in rodent models. (24)

The most striking reduction in risk with prucalopride was not for depression, but for psychotic disorders. However, it is a *post hoc* finding that we did not hypothesise, and the very low incidence and wide confidence intervals lead us to interpret this finding, as for bipolar disorder, with great caution. Moreover, in contrast to depression, the Kaplan-Meier curves for psychotic disorder show earlier separation between cohorts for the comparison with linaclotide, increasing the likelihood that the findings are related to unmeasured confounding. Nevertheless, the potential pro-cognitive profile of 5-HT_4_R agonism may be a transdiagnostic mechanism through which prucalopride lowers the risk for both depression *and* other psychiatric disorders. Thus, the possibility of benefits of 5-HT_4_R agonism on psychosis merits investigation in additional, and younger, cohorts.

In emulated target trials, it is important to consider whether there are systematic differences between the groups exposed to one drug vs. another, which may confound the findings. Importantly, the three drugs compared in this study have the same clinical indication and the cohorts were well matched on a wide range of covariates at baseline. With E-values of 1.58 and 1.83 for the main analyses, any residual confounder would need to be associated with both the exposure and the outcome with a relative risk of 1.58-1.83 to explain away the observed association. Furthermore, results from the robustness analyses argue against the possibility of such large residual confounding. For example, replication after excluding those with a recent prescription of the most commonly used SSRIs (often prescribed for menopause, and irritable bowel syndrome) eliminates possible differential impact of these drugs on outcomes. The absence of a difference in depression incidence between other anti-constipation agents with difference in prices and Medicaid/Medicare approval status suggests that those factors were not driving the observed differences. The overlap between Kaplan-Meier curves in the early phase of follow-up argues against obvious selection bias which can manifest as effects that appear too early. (25) Finally, the findings from interrupted time series analysis provide further evidence that initiation of prucalopride may decrease the risk of depression, and complements the emulated target trial design: the former benefits from better control of unmeasured time-invariant covariates while the latter distinguishes the effect of 5-HT_4_R agonism from a generic anti-constipation effect.

Our study has several strengths, including a large sample size, propensity score matching for a wide range of covariates, and consistency of findings across robustness analyses. However, it also has limitations. Some are generic to studies based on electronic health records including coding errors, patients receiving care outside the network, and the uncertain compliance with medications (which is why this study emulates an intention-to-treat analysis). There are also limitations specific to this study. First, it is possible that the effect of prucalopride on depression is partly mediated by better control of constipation than comparator drugs. However, network meta-analyses have shown no significant differences in efficacy between these drugs in the treatment of chronic idiopathic constipation (26) and opioid-induced constipation, (27) and enema use was similar between cohorts. Second, we could not estimate robust variances accounting for the same individuals being potentially present in two cohorts (if a patient received both prucalopride and one of the comparator drugs at two different time points) since identification of patients across cohorts could threaten anonymity. However, results were robust when mutually exclusive cohorts were used (for which the variance estimates of this study are valid) and after excluding patients who crossed over to the other cohort during follow-up, suggesting that variance estimates did not result in false positive findings. This is also supported by the minimal overlap between prucalopride and linaclotide/lubiprostone cohorts (Supplementary Figure 3). Other limitations include uncertainty about whether findings generalise to patients without constipation or in countries outside the US; sample sizes were insufficient to stratify analyses by sex or age; and a diagnosis of chronic constipation could not be confirmed in all included participants in the cohorts as it is notoriously undercoded in electronic health records. (28) Some patients might have therefore received these drugs off-label for other indications. Finally, it is possible that clinicians may be more cautious of prucalopride in patients potentially at risk of mental illness due to early FDA warnings of a risk of increased suicidal thoughts or behaviour. The focus on first episode of depression in people with no history of mental illness mitigates this risk. In addition, people with a history of mental illness are actually *more* likely to be prescribed prucalopride than either comparator drug (Supplementary Table 16).

Despite the exclusion of people with a previous diagnosis of common and/or serious mental illness in our primary analysis, antidepressant use in our sample is high (see Table 1 and Supplementary Tables 3-5). This is likely due to the common usage of antidepressants, especially non-SSRIs, for reasons other than depression (or anxiety) in people with chronic gastrointestinal illness, such as pain and urinary symptoms. Excluding all people with a history or concurrent use of antidepressants would therefore challenge the external validity of our findings. To address any bias due to differences in use of antidepressants, these were included as covariates (both as a class and as individual agents) and matched for. Their large prevalence in the cohorts leaves the possibility that the observed associations reflect a synergistic effect between 5-HT_4_R agonists and antidepressants, as suggested by pre-clinical evidence. (29)

This emulated target trial provides robust clinical evidence that prucalopride is associated with a lower risk of depression in those with no previous diagnosis compared to alternative anti-constipation agents, supporting the hypothesis that 5-HT_4_R agonists have antidepressant properties. This evidence lends strong support for further investigation of the effect of 5-HT_4_R agonists on depression within a randomised controlled trial, and also consideration for its use within other mental illnesses.

## Supplementary Material

Supplementary material

## Figures and Tables

**Table 1:** Baseline characteristics of matched cohorts of patients receiving prucalopride vs. linaclotide (left columns) or prucalopride vs. lubiprostone (right columns) Lifetime prevalences are given as percentages. Baseline characteristics with a prevalence of at least 5% were included. All characteristics used for matching can be found in Supplementary Table 2

	COMPARISON 1		COMPARISON 2	
	Prucalopride	Linaclotide	Prucalopride	Lubiprostone
*Cohort size (n)*	8572	8572	8281	8281
*Age, years, mean (SD)*	49.0 (18.9)	48.7 (18.7)	49.1 (18.9)	48.7 (18.8)
*Sex (M, F, unknown / other)*	20.5, 75.9, 3.6	19.9, 76.3, 3.8	20.7, 75.6, 3.7	19.7, 76.2, 4.1
*Ethnicity (White, Black, unknown / other)*	71.7, 9.6, 18.7	73.2, 8.2, 18.6	71.7, 9.7, 18.6	71.6, 9.2, 19.2
*Marital status (married / partner, divorced / separated, widowed, never married, unknown)*	20.6, 3.6, 2.5, 12.4, 61.1	19.9, 3.6, 2.6, 12.2, 61.9	20.8, 3.7, 2.6, 12.7, 60.2	20.2, 3.6, 2.6, 12.4, 61.2
*COMORBIDITIES, %*				
*Gastro-oesophageal reflux disease*	35.2	34.7	34.1	34.1
*Primary hypertension*	20.3	19.6	20.3	19.2
*IBS*	19.1	19.3	18.8	19.7
*Lipidaemias*	18.7	18.6	18.6	18.5
*Disorders of thyroid gland*	13.9	13.4	13.7	14.2
*Diabetes mellitus*	11.9	11.9	11.6	11.7
*Chronic pain*	13.3	13.2	13.0	13.6
*Overweight, obesity*	10.1	10.0	9.8	10.2
*Ischaemic heart diseases*	7.4	7.3	7.3	7.2
*Acute and chronic kidney disease*	5.5	5.2	5.5	5.3
*Digestive malignancy*	1.1	1.1	1.2	1.1
*Demyelinating diseases of the nervous system*	1.5	1.6	1.5	1.6
*Parkinsons disease*	0.9	0.8	0.9	0.9
*MEDICATIONS, %*				
*Laxatives*	54.1	54.2	54.0	54.2
*Opioid analgesics*	49.8	49.3	49.6	49.0
*Sedatives / hypnotics*	47.1	47.3	46.9	46.8
*Corticosteroids*	47.8	47.2	47.4	47.8
*Antidepressants*	40.6	40.7	40.3	40.7
*Antilipaemic agents*	26.5	25.6	26.6	26.2
*Stimulant laxatives*	30.4	30.5	30.3	30.6
*Stool softeners*	19.6	19.8	19.7	19.6
*Thyroid modifiers*	16.2	15.3	16.2	16.0
*Antipsychotics*	8.9	8.9	8.8	8.1
*Duloxetine*	9.0	9.1	8.8	8.8
*Trazodone*	8.2	8.2	8.2	8.3
*Sertraline*	5.8	4.9	4.8	4.9
*Escitalopram*	5.3	5.2	5.2	5.5
*Mirtazapine*	4.6	4.3	4.1	3.9
*Fluoxetine*	3.3	3.3	3.4	3.5
*Citalopram*	2.4	2.5	2.4	2.6

**Table 2:** Results for secondary outcomes comparing matched cohorts of individuals prescribed prucalopride vs. linaclotide / lubiprostone Incidences reported at 1 year. Bold values for hazard ratios & p-values indicate statistical significance. The proportional hazard assumption (PHA) is rejected if p<0.05 (Supplementary Figure 4).

	Prucalopride incidence (%)	Linaclotide incidence (%)	Hazard ratio (95% CI)	P values	E values	PHA
*Mood disorder (F30-39)*	7.07 (6.47-7.72)	8.34 (7.71-9.02)	**0.85 (0.75-0.96)**	**0.0074**	1.64	0.43
*Bipolar disorder (F31)*	0.69 (0.51-0.92)	1.01 (0.80-1.28)	**0.69 (0.48-1.00)**	**0.048**	2.26	0.40
*Psychotic disorder (F20-29)*	0.082 (0.037-0.18)	0.31 (0.20-0.47)	**0.27 (0.11-0.67)**	**0.0019**	6.78	0.15
*Dementia (F01-F03, G30, G31.0, G31.2, G31.83)*	0.34 (0.22-0.52)	0.55 (0.40-0.76)	0.60 (0.36-1.02)	0.056	N/A	0.70
*Anxiety disorder (F40-48)*	10.23 (9.52-10.99)	10.71 (10.00-11.46)	0.96 (0.87-1.07)	0.49	N/A	0.17
*Substance use disorder (F10-19)*	2.57 (2.21-3.00)	3.11 (2.72-3.55)	**0.80 (0.65-0.98)**	**0.033**	1.81	**0.02**
*Any of negative control outcomes*	3.39 (2.94-3.91)	3.14 (2.73-3.61)	1.05 (0.86-1.28)	0.66	N/A	**0.02**
	***Prucalopride incidence (%)***	***Lubiprostone incidence (%)***	***Hazard ratio (95% CI)***	***P values***	***E values***	***PHA***
*Mood disorder (F30-39)*	6.89 (6.29-7.55)	8.55 (7.91-9.24)	**0.81 (0.71-0.91)**	**0.00054**	1.79	0.36
*Bipolar disorder (F31)*	0.67 (0.49-0.90)	0.77 (0.59-1.01)	0.91 (0.61-1.36)	0.64	N/A	**0.05**
*Psychotic disorder (F20-29)*	0.072 (0.03-0.17)	0.29 (0.19-0.45)	**0.26 (0.096-0.68)**	**0.0023**	7.27	0.16
*Dementia (F01-F03, G30, G31.0, G31.2, G31.83)*	0.33 (0.21-0.51)	0.49 (0.35-0.69)	0.67 (0.38-1.16)	0.15	N/A	0.81
*Anxiety disorder (F40-48)*	10.20 (9.47-10.97)	10.56 (9.86-11.31)	0.98 (0.88-1.09)	0.75	N/A	**0.013**
*Substance use disorder (F10-19)*	2.62 (2.24-3.06)	2.78 (2.40-3.21)	0.94 (0.76-1.17)	0.59	N/A	0.98
*Any of negative control outcomes*	3.38 (2.92-3.91)	3.80 (3.35-4.32)	0.88 (0.72-1.07)	0.19	N/A	0.39

## Data Availability

As described in the manuscript methods, the TrinetX US Collaborative Network was used for this study. This is a cloud-based network that can access anonymised data from electronic health records in multiple healthcare organisations, in the United States (US). Each healthcare organisation remains anonymous within TriNetX, and is able to provide data with the necessary consents and approvals as long as research is the sole use. Data de-identification is formally attested as per Section §164.514(b)(1) of the HIPAA Privacy Rule, superseding TriNetX’s waiver from the Western Institutional Review Board; no further ethical approval was thus needed. As we used anonymised routinely collected data, no participant consent was required. The TriNetX system returned the results of these analyses as csv files, which we downloaded and archived. Aggregate data, as presented in this article, can be freely accessed in the Open Science Framework at https://osf.io/zqf4s/ [data will be uploaded upon acceptance of the article]. The data used for this article were acquired from TriNetX. This study had no special privileges. Inclusion criteria specified in the methods would allow other researchers to identify similar cohorts of patients as we used here for these analyses; however, TriNetX is a live platform with new data being added daily so exact counts will vary. To gain access to the data, a request can be made to TriNetX (join@trinetx.com), but costs might be incurred, and a data sharing agreement would be necessary.

## Relevance Statement

Many patients do not achieve remission with current antidepressants and treatments with novel mechanisms of action are needed. In this emulated target trial, we provide the first clinical evidence for the potential effectiveness of 5-HT_4_ receptor agonists against depression, which supports their further investigation in randomised controlled trials. These findings suggest that 5-HT_4_ receptor agonists might become part of the clinician’s toolbox to prevent or treat depression in the future.

## Analytic code availability

Analysis code for this article was produced using the “survival” package in the open source platform, R 4.2.1, adapted from code used in previous publications. That is authors estimated cumulative incidences over the one-year follow-up using the Kaplan-Meier estimator. The Cox proportional hazard model and the log-rank test were used to compare matched cohorts in terms of hazard ratios (HR) with 95% confidence intervals (CI). We also tested the proportional hazards assumption and calculates E-values for all comparisons. The code is openly accessible to any reviewer upon request.
