# Association between a selective 5-HT_4_ receptor agonist and incidence of major depressive disorder: emulated target trial

**DOI:** 10.1192/bjp.2024.97

**Published:** 2024-09

**Authors:** Angharad N. de Cates, Catherine J. Harmer, Paul J. Harrison, Philip J. Cowen, Anton Emmanuel, Simon Travis, Susannah E. Murphy, Maxime Taquet

**Affiliations:** Department of Psychiatry, Warneford Hospital, University of Oxford, UK; and Institute for Mental Health, University of Birmingham, UK; Department of Psychiatry, Warneford Hospital, University of Oxford, UK; Warneford Hospital, Oxford Health NHS Foundation Trust, Oxford, UK; and Oxford Centre for Human Brain Activity and Oxford Centre for Functional MRI of the Brain, Wellcome Centre for Integrative Neuroimaging, Department of Psychiatry, University of Oxford, UK; Department of Psychiatry, Warneford Hospital, University of Oxford, UK; and Warneford Hospital, Oxford Health NHS Foundation Trust, Oxford, UK; GI Physiology Unit, University College London Hospitals NHS Foundation Trust, London, UK; Kennedy Institute of Rheumatology, University of Oxford, UK; and Translational Gastroenterology and Liver Unit, University of Oxford, UK

**Keywords:** Electronic health records, big data, survival analysis, depressive disorders, antidepressants

## Abstract

**Background:**

The serotonin 4 receptor (5-HT_4_R) is a promising target for the treatment of depression. Highly selective 5-HT_4_R agonists, such as prucalopride, have antidepressant-like and procognitive effects in preclinical models, but their clinical effects are not yet established.

**Aims:**

To determine whether prucalopride (a 5-HT_4_R agonist and licensed treatment for constipation) is associated with reduced incidence of depression in individuals with no past history of mental illness, compared with anti-constipation agents with no effect on the central nervous system.

**Method:**

Using anonymised routinely collected data from a large-scale USA electronic health records network, we conducted an emulated target trial comparing depression incidence over 1 year in individuals without prior diagnoses of major mental illness, who initiated treatment with prucalopride versus two alternative anti-constipation agents that act by different mechanisms (linaclotide and lubiprostone). Cohorts were matched for 121 covariates capturing sociodemographic factors, and historical and/or concurrent comorbidities and medications. The primary outcome was a first diagnosis of major depressive disorder (ICD-10 code F32) within 1 year of the index date. Robustness of the results to changes in model and population specification was tested. Secondary outcomes included a first diagnosis of six other neuropsychiatric disorders.

**Results:**

Treatment with prucalopride was associated with significantly lower incidence of depression in the following year compared with linaclotide (hazard ratio 0.87, 95% CI 0.76–0.99; *P* = 0.038; *n* = 8572 in each matched cohort) and lubiprostone (hazard ratio 0.79, 95% CI 0.69–0.91; *P* < 0.001; *n* = 8281). Significantly lower risks of all mood disorders and psychosis were also observed. Results were similar across robustness analyses.

**Conclusions:**

These findings support preclinical data and suggest a role for 5-HT_4_R agonists as novel agents in the prevention of major depression. These findings should stimulate randomised controlled trials to confirm if these agents can serve as a novel class of antidepressant within a clinical setting.

Depression is a leading cause of disability worldwide, and improving its treatment is a global health priority.^1^ A third of patients do not achieve remission with current treatment approaches, which has a significant effect on occupational and social functioning.^2^ First-line antidepressants also have limited efficacy in treating some symptom clusters, such as cognitive impairment.^3^ Within this context, there is a pressing need for novel antidepressant targets.^4^ However, drug discovery in psychiatry is notoriously challenging, with fewer than half of the new drugs approved by the USA Food and Drug Administration (FDA) in the past 5 years in neurology.^5^ One promising strategy is the repurposing of drugs known to act on receptors in the central nervous system and used to treat conditions affecting other organ systems.^6^

A first step in drug repurposing is the identification of a candidate compound. Serotonin 4 postsynaptic receptors (5-HT_4_Rs) are widely expressed in the brain, particularly in regions and networks related to mood and cognitive functioning (see Fig. 1).^7^ 5-HT_4_R agonists have rapid antidepressant-like effects in animal models of depression,^8,9^ and a facilitatory effect on behavioural tasks of learning and memory in rodents.^10^ In humans, there is also emerging experimental evidence to support a role for the 5-HT_4_R in depression and cognition.^11,12^ Brain 5-HT_4_R binding is reduced in patients with depression compared with healthy controls, and associated with deficits in memory performance.^11^ However, clinical evidence for the effect of 5-HT_4_R agonists on depression is lacking. Emulated target trials allow assessment of the effect of a treatment on a clinical outcome outside of the existing license, using observational data.^13^ Using this approach, we assessed whether exposure to the 5-HT_4_R agonist prucalopride (an anti-constipation agent acting on 5-HT_4_R in the gut, but also with good brain penetration^14^) is associated with a lower risk of depression within 1 year since first prescription, compared with two alternative anti-constipation agents that have no known effect on 5-HT_4_Rs or the central nervous system. We hypothesised that participants on prucalopride would show a lower risk of depression within 1 year.

**Fig. 1:**
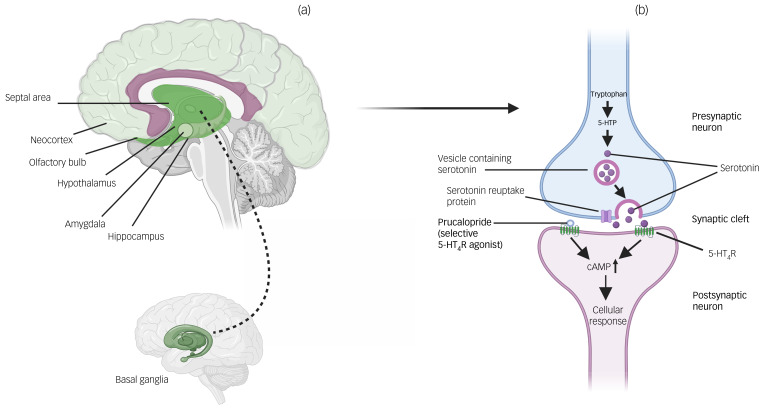
Locations of 5-HT_4_Rs in the brain and actions of prucalopride. (a) Brain regions where 5-HT_4_Rs are particularly highly expressed (see Beliveau et al^7^). Darker shading indicates regions of highest expression (i.e. basal ganglia); lighter shading indicates relatively lower expression (i.e. neocortex). (b) Action of prucalopride at a serotonin synapse as a highly selective agonist at transmembrane G-protein-coupled 5-HT_4_Rs. 5-HT_4_R, serotonin 4 receptor; 5-HTP, 5-hydroxytryptophan; cAMP, cyclic adenosine monophosphate.

## Method

### Study design and data collection

The TriNetX USA Collaborative Network was used for this study, which is a network of anonymised electronic health records data from 60 healthcare organisations in the USA.^15^ In brief, this network allows access to over 100 million patients, with data including demographics, diagnoses and ICD-10 codes, medications, procedures, measurements (e.g. blood pressure, body mass index) and healthcare visits (see Supplementary Methods 1 available at https://doi.org/10.1192/bjp.2024.97 for details). Data is included from both primary and specialist healthcare organisations, and involves both patients insured and not insured under the standard USA system. Each healthcare organisation remains anonymous within TriNetX, and is able to provide data with the necessary consents and approvals as long as research is the sole use. The process of data de-identification is attested by a qualified expert as defined in Section 164.514(b)^1^ of the Health Insurance Portability and Accountability Act of 1996 (HIPAA) Privacy Rule.^16^ No further ethical approval is needed. As we used anonymised routinely collected data, no participant consent was required. We followed the approach of Hernán and Robins to emulate a target trial using electronic health records from TriNetX.^13^ The different components of the target and emulated trials are summarised below and detailed in the Supplementary Methods and Supplementary Table 1. We followed the Strengthening the Reporting of Observational Studies in Epidemiology (STROBE) guidelines (see Supplementary Materials).

### Participants and exposure

The primary cohort included individuals with a first prescription of prucalopride before the start date of analysis (25 January 2024). Two comparator cohorts were defined as individuals with a first prescription of linaclotide (a guanylate cyclase 2C agonist) or lubiprostone (a chloride channel activator) before 25 January 2024. For all cohorts, no age or gender restriction was applied, but patients with pre-existing diagnoses of common and/or serious mental illness were excluded (ICD-10 codes for psychotic disorders (F20–F29), mood disorders (F30–F39), anxiety disorders (F40–F48) and mental disorders due to known physiological conditions including cognitive disorders (F01-F09)). Patients were censored at their last clinical encounter or when they died. An intention-to-treat analysis was emulated by including all individuals who received one prescription of the drug in the corresponding cohort. See Supplementary Methods 2 for details on cohort definition.

All three medications (prucalopride, linaclotide and lubiprostone) are approved by the FDA for use in the USA for chronic constipation, are third-line pharmacotherapy options (i.e. when laxatives alone are insufficient) in national guidelines^17^ and are available under Medicaid/Medicare.

### Covariates

Cohorts were matched with propensity score matching for 121 variables capturing sociodemographic factors, and concurrent or history of comorbidities and medications that could be associated with differences in choices of anti-constipation treatment and/or with psychiatric disorders. These covariates were selected based on expert opinion as well as statistical differences between cohorts before matching (see Supplementary Methods 3 for details and Supplementary Table 2). Baseline characteristics of cohorts before matching (lifetime, 5 years and 1 year before inclusion) were checked (Supplementary Tables 3–5).

### Outcomes

The primary outcome was the incidence of a first diagnosis of major depressive disorder (F32) within 1 year of the index date. Secondary outcomes included incidence of a first diagnosis of six other common and/or serious neuropsychiatric disorders in the first year: (a) mood (affective) disorder (F30–F39), as an overarching outcome, as well as (b) bipolar disorder (F31) specifically; (c) anxiety disorder (F40–F48); (d) dementia (any of F01–F03, G30, G31.0, G31.2 or G31.83); (e) substance use disorder (F10–F19) and (f) psychotic disorder (F20–F29).

### Secondary analyses

Details on all secondary analyses can be found in Supplementary Methods 5.

#### Robustness analyses

Robustness of the results to changes in outcome, cohort and model specifications was tested under the following conditions: (a) excluding individuals with a contraindication for any of the study medications from all cohorts; (b) excluding those within the comparator (linaclotide/lubiprostone) cohort who had a prescription of prucalopride in the year before the index date, thus making cohorts mutually exclusive; (c) excluding those with a recorded prescription of the alternate drug in the 1 year following the index date from each cohort; (d) excluding those with additional pre-existing neuropsychiatric disorders and (e) excluding patients with a recent prescription of either of the two most common selective serotonin reuptake inhibitor (SSRI) antidepressants (escitalopram and sertraline).

#### Negative control outcomes

To assess for potential unmeasured confounding, matched cohorts were compared in terms of a range of negative control outcomes that are not expected to be influenced by differences in anti-constipation medications.^18^ Twenty negative control outcomes were selected, adapted from previous analyses using the TriNetX database,^19^ and first occurrences analysed both individually and as a composite outcome. A full list of negative control outcomes is available in the Supplementary Methods.

#### Additional comparisons

Influences of medication costs and Medicaid/Medicare availability were tested by using additional comparisons (in terms of psychiatric and negative control outcomes): (a) linaclotide (similar price as prucalopride) versus lubiprostone (cheaper than prucalopride) and (b) plecanatide (same mechanism of action as linaclotide and available under Medicaid/Medicare at the same time as prucalopride) versus linaclotide (available under Medicaid/Medicare earlier than prucalopride).

To assess whether differences in incidence of depression could be explained by differences in efficacy of anti-constipation drugs, we compared prucalopride with both comparator drugs in terms of incidence of an enema (taken to be an indicator of suboptimal anti-constipation treatment) over the 1-year follow-up period.

#### Interrupted time-series analysis

We complemented the emulated target trial with an interrupted time-series analysis comparing the trend in depression incidence in the 12 months after versus before the first prescription of prucalopride, and relative to the total number of people who had at least one health encounter within each month. We hypothesised a change in slope, with a progressive reduction in the number of depression diagnoses after initiation of prucalopride. This analysis was also conducted for negative control outcomes.

### Statistical analysis

Propensity score 1:1 matching was achieved with a greedy nearest neighbour algorithm with a calliper distance of 0.1. Matching for a covariate was considered adequate if the standardised mean difference between matched cohorts was <0.1.^15^ Cumulative incidences over the 1-year follow-up period were estimated with the Kaplan–Meier estimator. The log-rank test was used to compare survival between matched cohorts and the Cox proportional hazard model was used to estimate hazard ratios with 95% confidence intervals (using the ‘survival’ package in R, version 4.2.1 for Windows; https://www.r-project.org/). The proportional hazard assumption was tested with the generalised Schoenfeld approach. E-values were calculated for all comparisons in the primary analysis. ^20^ Statistical significance was set at a two-sided *P* < 0.05.

## Results

A total of 8694 patients had at least one prescription of prucalopride (mean age 48.8, s.d. 19.0, 75.8% female, 20.6% male), of which 8572 and 8281 were successfully matched 1:1 to patients with a first prescription of linaclotide or lubiprostone, respectively (see Table 1 for baseline characteristics of matched cohorts and Supplementary Tables 3–5 for baseline characteristics before matching).

**Table 1:** Baseline characteristics of matched cohorts of patients receiving prucalopride versus linaclotide (left columns) or prucalopride versus lubiprostone (right columns)

	Comparison 1		Comparison 2	
	Prucalopride	Linaclotide	Prucalopride	Lubiprostone
Cohort size, *n*	8572	8572	8281	8281
Age, years, mean (s.d.)	49.0 (18.9)	48.7 (18.7)	49.1 (18.9)	48.7 (18.8)
Gender (male, female, unknown/other)	20.5, 75.9, 3.6	19.9, 76.3, 3.8	20.7, 75.6, 3.7	19.7, 76.2, 4.1
Ethnicity (White, Black, unknown/other)	71.7, 9.6, 18.7	73.2, 8.2, 18.6	71.7, 9.7, 18.6	71.6, 9.2, 19.2
Marital status (married/partner, divorced/separated, widowed, never married, unknown)	20.6, 3.6, 2.5, 12.4, 61.1	19.9, 3.6, 2.6, 12.2, 61.9	20.8, 3.7, 2.6, 12.7, 60.2	20.2, 3.6, 2.6, 12.4, 61.2
Comorbidities, %				
Gastro-oesophageal reflux disease	35.2	34.7	34.1	34.1
Primary hypertension	20.3	19.6	20.3	19.2
Irritable bowel syndrome	19.1	19.3	18.8	19.7
Lipidaemias	18.7	18.6	18.6	18.5
Disorders of thyroid gland	13.9	13.4	13.7	14.2
Diabetes mellitus	11.9	11.9	11.6	11.7
Chronic pain	13.3	13.2	13.0	13.6
Overweight, obesity	10.1	10.0	9.8	10.2
Ischaemic heart diseases	7.4	7.3	7.3	7.2
Acute and chronic kidney disease	5.5	5.2	5.5	5.3
Digestive malignancy	1.1	1.1	1.2	1.1
Demyelinating diseases of the nervous system	1.5	1.6	1.5	1.6
Parkinson's disease	0.9	0.8	0.9	0.9
Medications, %				
Laxatives	54.1	54.2	54.0	54.2
Opioid analgesics	49.8	49.3	49.6	49.0
Sedatives/hypnotics	47.1	47.3	46.9	46.8
Corticosteroids	47.8	47.2	47.4	47.8
Antidepressants	40.6	40.7	40.3	40.7
Antilipaemic agents	26.5	25.6	26.6	26.2
Stimulant laxatives	30.4	30.5	30.3	30.6
Stool softeners	19.6	19.8	19.7	19.6
Thyroid modifiers	16.2	15.3	16.2	16.0
Antipsychotics	8.9	8.9	8.8	8.1
Duloxetine	9.0	9.1	8.8	8.8
Trazodone	8.2	8.2	8.2	8.3
Sertraline	5.8	4.9	4.8	4.9
Escitalopram	5.3	5.2	5.2	5.5
Mirtazapine	4.6	4.3	4.1	3.9
Fluoxetine	3.3	3.3	3.4	3.5
Citalopram	2.4	2.5	2.4	2.6

Lifetime prevalences are given as percentages. Baseline characteristics with a prevalence of at least 5% were included. All characteristics used for matching can be found in Supplementary Table 2.

**Fig. 2:**
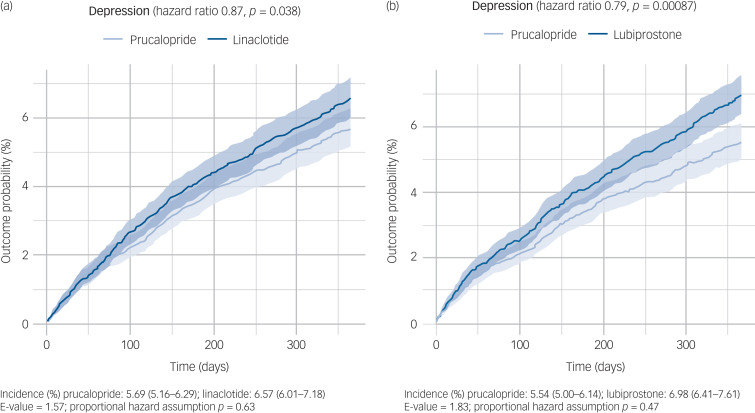
Kaplan–Meier curves showing the cumulative incidence of depression diagnosis over 12 months in those receiving (a) prucalopride versus linaclotide and (b) prucalopride versus lubiprostone. The shaded areas around curves represent 95% CI.

In the year following a first prescription, those prescribed prucalopride had a significantly lower incidence of depression (prucalopride versus linaclotide: hazard ratio 0.87, 95% CI 0.76–0.99, *P* = 0.038, E = 1.58; prucalopride versus lubiprostone: hazard ratio 0.79, 95% CI 0.69–0.91, *P* < 0.001, E = 1.83; Fig. 2). There was no evidence of non-proportional hazards for either comparison (*P* = 0.63 and 0.47, respectively).

These results were replicated in all robustness analyses for lubiprostone, and all but one for linaclotide (Supplementary Table 6). In particular, the findings held when we excluded participants who had a contraindication for any drug being compared, patients who crossed over to the other ‘arm’ of the comparison within the 1-year follow-up period or patients who had a recent prescription of one of the two most common SSRI antidepressants. The only exception was the comparison with linaclotide when participants with a broader range of neuropsychiatric diagnoses were excluded, which resulted in a non-significant association of similar effect size (hazard ratio 0.87, 95% CI 0.76–1.00, *P* = 0.058).

In terms of secondary outcomes (Table 2 and Supplementary Figs 1 and 2), compared with linaclotide and lubiprostone, those prescribed prucalopride had a lower incidence of all mood disorders (prucalopride versus linaclotide: hazard ratio 0.85, 95% CI 0.75–0.96, *P* = 0.0074; prucalopride versus lubiprostone: hazard ratio 0.81, 95% CI 0.71–0.91, *P* < 0.001) and psychotic disorder (prucalopride versus linaclotide: hazard ratio 0.27, 95% CI 0.11–0.67, *P* = 0.0019; prucalopride versus lubiprostone: hazard ratio 0.26, 95% CI 0.096–0.68, *P* = 0.0023). Results for these two secondary outcomes were replicated in all robustness analyses (Supplementary Tables 7–11). Findings for other outcomes, including dementia, bipolar disorder and substance misuse, varied in significance across analyses.

**Table 2:** Results for secondary outcomes comparing matched cohorts of individuals prescribed prucalopride versus linaclotide/lubiprostone

	Incidence after prucalopride (%)	Incidence after linaclotide (%)	Hazard ratio (95% CI)	*P*-values	E-values	PHA
Mood disorder (F30–39)	7.07 (6.47–7.72)	8.34 (7.71–9.02)	**0.85 (0.75–0.96)**	**0.0074**	1.64	0.43
Bipolar disorder (F31)	0.69 (0.51–0.92)	1.01 (0.80–1.28)	**0.69 (0.48–1.00)**	**0.048**	2.26	0.40
Psychotic disorder (F20–29)	0.082 (0.037–0.18)	0.31 (0.20–0.47)	**0.27 (0.11–0.67)**	**0.0019**	6.78	0.15
Dementia (F01–F03, G30, G31.0, G31.2, G31.83)	0.34 (0.22–0.52)	0.55 (0.40–0.76)	0.60 (0.36–1.02)	0.056	Not applicable	0.70
Anxiety disorder (F40–48)	10.23 (9.52–10.99)	10.71 (10.00–11.46)	0.96 (0.87–1.07)	0.49	Not applicable	0.17
Substance use disorder (F10–19)	2.57 (2.21–3.00)	3.11 (2.72–3.55)	**0.80 (0.65–0.98)**	**0.033**	1.81	**0.02**
Any of negative control outcomes	3.39 (2.94–3.91)	3.14 (2.73–3.61)	1.05 (0.86–1.28)	0.66	Not applicable	**0.02**

	Incidence after prucalopride (%)	Incidence after lubiprostone (%)	Hazard ratio (95% CI)	*P*-values	E-values	PHA
Mood disorder (F30–39)	6.89 (6.29–7.55)	8.55 (7.91–9.24)	**0.81 (0.71–0.91)**	**0.00054**	1.79	0.36
Bipolar disorder (F31)	0.67 (0.49–0.90)	0.77 (0.59–1.01)	0.91 (0.61–1.36)	0.64	Not applicable	**0.05**
Psychotic disorder (F20–29)	0.072 (0.03–0.17)	0.29 (0.19–0.45)	**0.26 (0.096–0.68)**	**0.0023**	7.27	0.16
Dementia (F01–F03, G30, G31.0, G31.2, G31.83)	0.33 (0.21–0.51)	0.49 (0.35–0.69)	0.67 (0.38–1.16)	0.15	Not applicable	0.81
Anxiety disorder (F40–48)	10.20 (9.47–10.97)	10.56 (9.86–11.31)	0.98 (0.88–1.09)	0.75	Not applicable	**0.013**
Substance use disorder (F10–19)	2.62 (2.24–3.06)	2.78 (2.40–3.21)	0.94 (0.76–1.17)	0.59	Not applicable	0.98
Any of negative control outcomes	3.38 (2.92–3.91)	3.80 (3.35–4.32)	0.88 (0.72–1.07)	0.19	Not applicable	0.39

Incidences reported at 1 year. Bold values for hazard ratios and *P*-values indicate statistical significance. The proportional hazard assumption (PHA) is rejected if *P* < 0.05 (Supplementary Fig. 4).

No significant differences in negative outcomes were observed (see Supplementary Tables 7–12). We also found no significant difference between matched cohorts in terms of risk of needing an enema (prucalopride versus linaclotide: hazard ratio 1.02, 95% CI 0.14–7.23, *P* = 0.99; prucalopride versus lubiprostone: hazard ratio 2.09, 95% CI 0.19–23.12, *P* = 0.53).

There was no significant difference in risk of depression between linaclotide and lubiprostone (*n* = 78 581 in each matched cohort; hazard ratio 1.00, 95% CI 0.95–1.04, *P* = 0.83), indicating that medication price is unlikely to confound the association (Supplementary Table 13; for rationale, see Method). Similarly, there was no significant difference in incidence of depression between linaclotide and plecanatide (*n* = 2405 patients in each matched cohort; hazard ratio 1.10, 95% CI 0.85–1.42, *P* = 0.46), indicating that Medicaid/Medicare availability date is unlikely to confound the association (Supplementary Table 14).

**Fig. 3:**
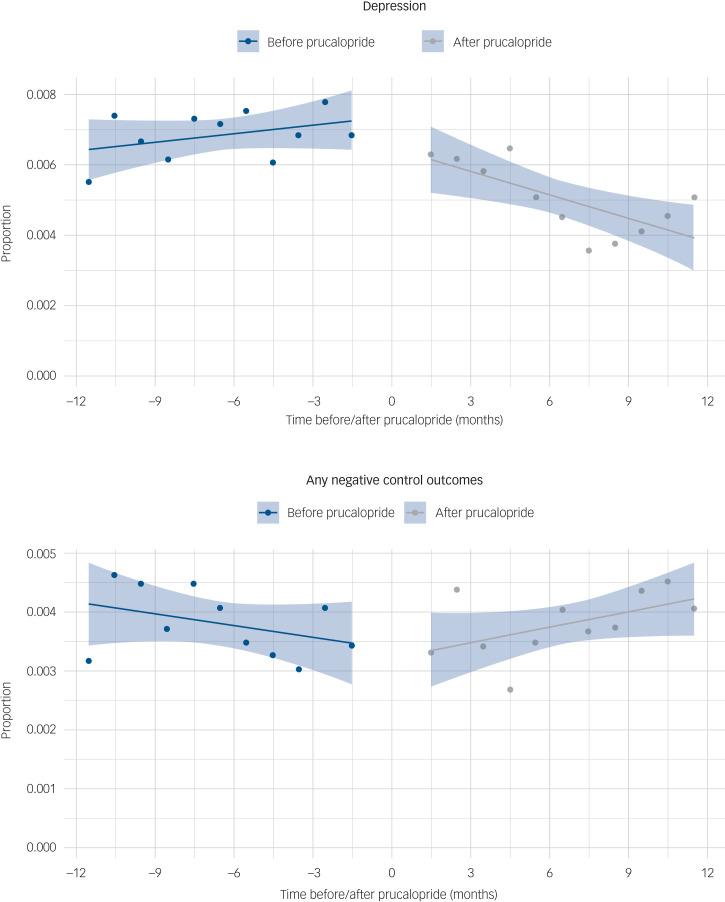
Interrupted time-series analysis comparing (a) the incidence of depression and (b) any negative control outcomes, before and after prucalopride prescription, shown as a proportion of people who had a healthcare encounter during each month.

In the interrupted time-series analysis, the incidence of depression significantly decreased after prescription of prucalopride (−3.05 cases/10 000 people per month, 95% CI −4.95 to −1.14, *P* = 0.0058; no evidence of autocorrelation, *P* = 0.63; Fig. 3(a)). This finding was robust when 2 months on either side of prucalopride prescription were included, and when absolute counts rather than incidence were analysed (Supplementary Table 15). Conversely, no decrease in incidence of negative control outcomes was observed over the follow-up period (+1.53 cases/10 000 people per month, 95% CI 0.12–2.93, *P* = 0.047; Fig. 3(b)).

## Discussion

In this emulated target trial, the highly selective 5-HT_4_R agonist, prucalopride, was associated with a 13–21% lower risk of a first episode of depression within the following year, compared with matched cohorts of patients prescribed drugs with similar indication but without action at the 5-HT_4_R (linaclotide and lubiprostone). This study is the first to consider the mental health effects of prucalopride by using clinical health records, and adds to a growing evidence base highlighting the potential role of 5-HT_4_R agonists in depression. It may also help to inform the choice of anti-constipation drug in people with chronic constipation who are at risk of depression.

The research question of this study lends itself well to an emulated target trial for two reasons: (a) all three drugs are third-line interventions for chronic constipation,^17^ and the choice between the three is largely driven by clinician's preference, thus limiting indication and selection bias; and (b) there is no *a priori* reason for patients and clinicians to have believed that these drugs would have differential effects on mental health, thereby limiting detection bias.

There are several mechanisms through which 5-HT_4_R agonism might reduce depression risk. First, 5-HT_4_R agonists may have a direct effect on mood after penetrating the blood–brain barrier, with evidence of increased brain derived neurotrophic factor/cyclic adenosine monophosphate response element binding protein production and direct action on the raphe nucleus in preclinical studies.^8,9^ Second, 5-HT_4_R agonists have procognitive effects in animal^10^ and human models of learning.^12,21^ Third, the association between prucalopride and depression may be mediated by action on the gut–brain axis, including effects on the microbiome or gut serotonin.^22^ Fourth, prucalopride may be acting to increase stress resilience: 5-HT_4_R agonists decrease stress-induced depressive-type behaviours in rodent models.^23^

The most striking reduction in risk with prucalopride was not for depression, but for psychotic disorders. However, it is a *post hoc* finding that we did not hypothesise, and the very low incidence and wide confidence intervals lead us to interpret this finding, as for bipolar disorder, with great caution. Moreover, in contrast to depression, the Kaplan–Meier curves for psychotic disorder show earlier separation between cohorts for the comparison with linaclotide, increasing the likelihood that the findings are related to unmeasured confounding. Nevertheless, the potential procognitive profile of 5-HT_4_R agonism may be a transdiagnostic mechanism through which prucalopride lowers the risk for both depression and other psychiatric disorders. Thus, the possibility of benefits of 5-HT_4_R agonism on psychosis merits investigation in additional, and younger, cohorts.

In emulated target trials, it is important to consider whether there are systematic differences between the groups exposed to one drug versus another, which may confound the findings. Importantly, the three drugs compared in this study have the same clinical indication, and the cohorts were well matched on a wide range of covariates at baseline. With E-values of 1.58 and 1.83 for the main analyses, any residual confounder would need to be associated with both the exposure and the outcome, with a relative risk of 1.58–1.83, to explain away the observed association. Furthermore, results from the robustness analyses argue against the possibility of such a large residual confounding. For example, replication after excluding those with a recent prescription of the most commonly used SSRIs (often prescribed for menopause and irritable bowel syndrome) eliminates a possible differential impact of these drugs on outcomes. The absence of a difference in depression incidence between other anti-constipation agents with difference in prices and Medicaid/Medicare approval status suggests that those factors were not driving the observed differences. The overlap between Kaplan–Meier curves in the early phase of follow-up argues against obvious selection bias, which can manifest as effects that appear too early.^24^ Finally, the findings from interrupted time-series analysis provide further evidence that initiation of prucalopride may decrease the risk of depression, and complements the emulated target trial design: the former benefits from better control of unmeasured time-invariant covariates, whereas the latter distinguishes the effect of 5-HT_4_R agonism from a generic anti-constipation effect.

Our study has several strengths, including a large sample size, propensity score matching for a wide range of covariates and consistency of findings across robustness analyses. However, it also has limitations. Some are generic to studies based on electronic health records, including coding errors, patients receiving care outside of the network and the uncertain adherence to medications (which is why this study emulates an intention-to-treat analysis). There are also limitations specific to this study. First, it is possible that the effect of prucalopride on depression is partly mediated by better control of constipation than comparator drugs. However, network meta-analyses have shown no significant differences in efficacy between these drugs in the treatment of chronic idiopathic constipation^25^ and opioid-induced constipation,^26^ and enema use was similar between cohorts. Second, we could not estimate robust variances accounting for the same individuals being potentially present in two cohorts (if a patient received both prucalopride and one of the comparator drugs at two different time points), since identification of patients across cohorts could threaten anonymity. However, results were robust when mutually exclusive cohorts were used (for which the variance estimates of this study are valid) and after excluding patients who crossed over to the other cohort during follow-up, suggesting that variance estimates did not result in false positive findings. This is also supported by the minimal overlap between prucalopride and linaclotide/lubiprostone cohorts (Supplementary Fig. 3). Other limitations include uncertainty about whether findings generalise to patients without constipation or in countries outside of the USA; sample sizes were insufficient to stratify analyses by gender or age; and a diagnosis of chronic constipation could not be confirmed in all included participants in the cohorts, as it is notoriously undercoded in electronic health records.^27^ Therefore, some patients might have received these drugs off-label for other indications. Finally, it is possible that clinicians may be more cautious of prucalopride in patients potentially at risk of mental illness, because of early FDA warnings of a risk of increased suicidal thoughts or behaviour. The focus on first episode of depression in people with no history of mental illness mitigates this risk. In addition, people with a history of mental illness are actually more likely to be prescribed prucalopride than either comparator drug (Supplementary Table 16).

Despite the exclusion of people with a previous diagnosis of common and/or serious mental illness in our primary analysis, antidepressant use in our sample is high (see Table 1 and Supplementary Tables 3–5). This is likely because of the common usage of antidepressants, especially non-SSRIs, for reasons other than depression (or anxiety) in people with chronic gastrointestinal illness, such as pain and urinary symptoms. Excluding all people with a history or concurrent use of antidepressants would therefore challenge the external validity of our findings. To address any bias from differences in use of antidepressants, these were included as covariates (both as a class and as individual agents) and matched for. Their large prevalence in the cohorts leaves the possibility that the observed associations reflect a synergistic effect between 5-HT_4_R agonists and antidepressants, as suggested by preclinical evidence.^28^

This emulated target trial provides robust clinical evidence that prucalopride is associated with a lower risk of depression in those with no previous diagnosis, compared with alternative anti-constipation agents, supporting the hypothesis that 5-HT_4_R agonists have antidepressant properties. This evidence lends strong support for further investigation of the effect of 5-HT_4_R agonists on depression within a randomised controlled trial, and consideration for its use within other mental illnesses.

## Supporting information

de Cates et al. supplementary material de Cates et al. supplementary material

## Data Availability

As described in the Method section, the TriNetX USA Collaborative Network was used for this study. This is a cloud-based network that can access anonymised data from electronic health records in multiple healthcare organisations, in the USA. Each healthcare organisation remains anonymous within TriNetX, and is able to provide data with the necessary consents and approvals as long as research is the sole use. Data de-identification is formally attested as per Section §164.514(b)^1^ of the HIPAA Privacy Rule, superseding TriNetX's waiver from the Western Institutional Review Board; no further ethical approval was thus needed. As we used anonymised routinely collected data, no participant consent was required. The TriNetX system returned the results of these analyses as csv files, which we downloaded and archived. Aggregate data, as presented in this article, can be freely accessed in the Open Science Framework at https://osf.io/zqf4s/. The data used for this article were acquired from TriNetX. This study had no special privileges. Inclusion criteria specified in the methods would allow other researchers to identify similar cohorts of patients as we used here for these analyses; however, TriNetX is a live platform with new data being added daily, so exact counts will vary. To gain access to the data, a request can be made to TriNetX (join@trinetx.com), but costs might be incurred, and a data-sharing agreement would be necessary. The analytic code used in this study is openly accessible to any reviewer, upon request to the corresponding author, A.N.d.C.
