# Supplementary material for: Association between a selective 5-HT4 receptor agonist and incidence of major depressive disorder: an emulated target trial
Source: Br J Psychiatry. Author manuscript; Available in PMC 2024 Sep 20. (PMC7616487; doi:10.1192/bjp.2024.97)
Supplement: Supplementary file 1 [file EMS196010-supplement-Supplementary_material.pdf]

de Cates et al.

Association between a selective 5-HT_4 receptor agonist and incidence of major depressive disorder: an emulated target trial

SUPPLEMENTARY MATERIAL

Contents:

Supplementary Methods 1-6
STROBE Checklist
Supplementary Tables 1 to 16
Supplementary Figures 1 to 4
Supplementary References

Supplementary Methods (Full)

Methods 1 - Study design and data collection

The TrinetX US Collaborative Network was used for this retrospective cohort study: a cloud-based network that can access anonymised data from electronic health records in multiple healthcare organisations in the United States (US). Full details of TriNetX can be found in (1, 2). In brief, this network allows access to over 100 million patients with data including demographics, diagnoses and ICD-10 codes, medications, procedures, measurements (e.g. blood pressure, body mass index) and health care visits. Data is included from both primary and specialist healthcare organisations, and involves both patients insured and not insured under the standard US system. Each healthcare organisation remains anonymous within TriNetX, and is able to provide data with the necessary consents and approvals as long as research is the sole use. The process of data de-identification is attested by a qualified expert as defined in Section 164.514(b)(1) of the HIPPA Privacy Rule (1). No further ethical approval is needed. As we used anonymised routinely collected data, no participant consent was required. We followed the approach of Hernán and Robins to emulate a target trial using electronic health records from TriNetX. (3) The different components of the target and emulated trials are detailed below and Supplementary Table 1. We followed STROBE guidelines (detailed in Supplementary Material pages 5-6).

Researchers use the TriNetX online interface to build various cohorts by inputting specific inclusion and exclusion criteria. Different cohorts can then be matched for confounding variables using propensity score matching. These cohorts can then be compared for outcomes of interest over a selected time frame.

Methods 2 - Cohorts

The primary cohort was defined as all patients with at least one (first) prescription of prucalopride before the start date of analysis (25th January 2024). Two comparator cohorts were also created using medications recommended for the same clinical purposes: patients with at least one (first) prescription of (i) linaclotide (a guanylate cyclase 2C agonist) and (ii) lubiprostone (a chloride channel activator). The index date (from which the follow-up started) was taken as the date of the first ever recorded prescription to avoid the bias of including prevalent users of study drugs. (4) An intention-to-treat analysis was emulated by including all individuals assigned to one cohort in the analysis even if they stopped taking the corresponding medication.

The cohorts included adults of any age or sex. Patients were censored at their last visit or when they died. Patients with pre-existing diagnoses of common and / or serious mental illness within their health records before the index date were excluded; that is with either code F20-F29 (schizophrenia, schizotypal, delusional, and other non-mood psychotic disorders), F30-F39 (mood [affective] disorders), F40-F48 (anxiety, dissociative, stress-related, somatoform and other nonpsychotic mental disorders), or F01-F09 (mental disorders due to known physiological conditions including cognitive disorders).

All three medications (prucalopride, linaclotide, and lubiprostone) are approved by the FDA for use in the United States for chronic constipation and all three drugs are available under Medicaid / Medicare. 5-HT_4 agonists, linaclotide and lubiprostone are third-line pharmacotherapy options in United States and other IBS-C guidelines (5, 6).

It is important to note that prucalopride, linaclotide and lubiprostone are all indicated for chronic constipation, and prucalopride is mentioned in UK irritable bowel syndrome (IBS) guidelines (6), but only linaclotide and lubiprostone have IBS as a specific indication. Nonetheless, matching for IBS achieved across analyses in this study guarantees that the prevalence of IBS was similar between cohorts. Ideally, we would also have selected people with the indication for these drugs (i.e. chronic constipation) coded (7-9).

Methods 3 - Covariates

All cohorts were matched for disorders and medications that could be associated with differences in choices of anti-constipation treatment and / or with psychiatric disorders according to expert opinion. In addition, matching occurred for any diagnoses or medication where the baseline incidence was greater than 5% in both cohorts and the difference between cohorts greater than 2% at the pre-matching stage. Propensity score matching was achieved within TriNetX using a greedy 1:1 nearest neighbour and a caliper of 0.1. Matching for a covariate was considered appropriate if a standardised mean difference of 0.1 or less was observed between matched cohorts (10). Baseline characteristics of cohorts prior to matching (lifetime, 5 years and 1 year before inclusion) were checked (Supplementary Tables 3-5).

This process resulted in matching for 121 variables including: age at index event, sex, race and ethnicity, marital status, body mass index/obesity, gastrointestinal (GI) diseases, metabolic disorders, diabetes mellitus, rheumatological disorders, neurological and neuropsychiatric disorders, cardiac and hypertensive disorders, endocrinological disorders, respiratory disorders, and GI and neuropsychiatric and pain medication. The list is detailed in Supplementary Material (Supplementary Table 2). Matching remained consistent across all analyses, except where otherwise stated.

Methods 4 - Outcomes

Primary outcome: The incidence of a first diagnosis of major depressive disorder (F32) within one year of the index date.

Secondary outcomes: The incidence of a first diagnosis of six other common and / or serious mental health outcomes (captured using the ICD-10 codes in brackets) within one year of the index date: (i) mood [affective] disorder (F30-F39), as an overarching outcome, as well as (ii) bipolar disorder (F31) specifically; (iii) anxiety disorder (F40-F48); (iv) dementia (any of F01-F03, G30, G31.0, G31.2, G31.83); (v) substance use disorder (F10-F19); (vi) psychotic disorder (F20-29).

Methods 5 - Secondary analyses

Robustness analyses

Robustness of the results to changes in outcome, cohort, and model specifications was tested under the following conditions: (i) excluding those who have a contraindication for a study medication (i.e. intestinal perforation [K63.1], intestinal obstruction [K56.601], inflammatory bowel disease [K50.9, K51.9], toxic megacolon [K59.31]); (ii) excluding those within the comparator cohort who have a prescription of prucalopride prior to the index date thus making cohorts mutually exclusive; (iii) excluding those with a recorded prescription of the alternate drug in the 1 year following the index date (i.e. in the prucalopride / linaclotide comparison, all those in the prucalopride cohort with linaclotide prescriptions during the year of follow-up, and those in the linaclotide cohort with prucalopride prescriptions during the year of follow-up were excluded); (iv) excluding those with additional pre-existing neuropsychiatric disorders (i.e. impulse disorders [F63]; extrapyramidal and movement disorders [G20-G26]; insomnias [F51.05 and F51.09]); (iv) excluding patients with pre-existing prescription of either of the two most common SSRI antidepressants (escitalopram and sertraline) in the six months prior to index date.

Negative control outcomes

To assess for potential unmeasured confounding, matched cohorts were also compared for a range of negative control outcomes, i.e. outcomes that are not expected to be influenced by differences in anti-constipation medications (11). 20 negative control outcomes were selected, adapted from previous similar analyses using the TriNetX database (12). These were designed to include both emergency and primary care, and various body systems and mechanisms: cutaneous abscess, ganglion, ingrowing nail, onycholysis, sebaceous cyst, senile keratosis, trigger finger, viral warts, insect bite, adhesive capsulitis of shoulder, blepharitis, fracture of hand, folliculitis, knee injury, paronychia, lateral epicondylitis (tennis elbow), otitis externa, nasal polyp, seborrheic dermatitis, and finger laceration. The incidence of a first diagnosis of each negative control outcome was assessed individually within a cohort, and also as a composite of any of these outcomes (to increase statistical power and thereby increase the chance of detecting small but significant effects of residual confounding).

Additional comparisons

The two anti-constipation comparators (linaclotide and lubiprostone) were compared with each other in terms of their effect on mental health and negative control outcomes to assess potential equivalence in incidence.

Prucalopride was licensed by the FDA more recently than linaclotide and lubiprostone in the US (prucalopride 2018; lubiprostone 2006; linaclotide 2012), and the prescription of prucalopride may be affected by social factors relating to Medicaid / Medicare availability. In order to control for this, linaclotide was compared with plecanatide. Plecanatide is a drug with the same mechanism of action and indication as linaclotide, and a similar Medicaid approval year as prucalopride: both prucalopride and plecanatide were approved for Medicaid / Medicare between 2018 and 2022, compared to several years earlier for linaclotide and lubiprostone (both available from 2014). To assess whether these factors played a role in the observed association, we ran additional analyses aiming to isolate the effect of these additional elements from the effect of 5-HT_4 agonism. Cohort limitations prevented direct comparison of prucalopride and plecanatide (there were too few participants

in each cohort to allow for them to be well-matched as a comparison). For analyses involving plecanatide, plecanatide use was not matched across cohorts.

Interrupted time series analysis
We complemented the emulated target trial analysis with an interrupted time series analysis comparing the trend in depression incidence in the 12 months after versus before the first prescription of prucalopride. The incidence was reported as the total number of cases of depression (N_d) relative to the total number of people who had at least one health encounter within each month (N_v). The linear regression used was:

$$I_d = \beta_0 + \beta_1 T + \beta_2 X_t + \beta_3 X_t T,$$

where $I_d = N_d/N_v$ is the incidence of depression measured each month, T is the time in months parameterised so that T=0 is the time of prescription of prucalopride, T=0.5, 1.5, 2.5, etc represent the time in months since prescription, and T=-0.5, -1.5, -2.5, etc represent the time in months before prescription, and Xt is a binary variable representing the period after (X_t=1) or before (X_t=0) prescription of prucalopride. The coefficient of interest was the change in slope β_3. We hypothesised a change in slope with a progressive reduction in the number of depression diagnoses after initiation of prucalopride. This analysis was also conducted for negative control outcomes.

Auto-correlation was assessed by examining the auto-correlation function and formally tested using the Breusch-Godfrey test. (13) Seasonality was not deemed relevant as time relative to prescription was used rather than calendar time. Incidences in the month just before and the month just after initiation of prucalopride were ignored to avoid possible contamination by increased depression incidence resulting from untreated constipation and to account for delays between prescription and initiation of treatment. We tested the robustness of the findings to inclusion of these two months, as well as to the use of absolute counts rather than relative incidences (i.e. N_d rather than N_d/N_v) in the time series (and using a quasi-Poisson link function in the regression).

Methods 6 - Statistical analysis
Cumulative incidences over the one-year follow-up were estimated using the Kaplan-Meier estimator. The log-rank test was used to compare survival between matched cohorts and the Cox proportional hazard model was used to estimate hazard ratios (HR) with 95% confidence intervals (CI) (computed using the "survival" package in R 4.2.1). The proportional hazard assumption was tested with the generalised Schoenfeld approach, and time-varying hazard ratios were calculated using natural cubic splines fitted to the log-cumulative hazard to identify where there was evidence of non-proportionality of hazards in the main comparison between the cohort of patients receiving prucalopride and a matched cohort receiving an alternative anti-constipation agent. E-values were calculated for all comparisons in the primary analysis. (14)

Statistical significance was set at two-sided $p<0.05$. Except for propensity score matching estimated within TriNetX, all statistical analyses were conducted using R version 4.2.1 from 25th January 2024.

STROBE Checklist

STROBE Statement—Checklist of items that should be included in reports of ***cohort studies***

	Item No	Recommendation	Page No
**Title and abstract**	1	(*a*) Indicate the study's design with a commonly used term in the title or the abstract<br>(*b*) Provide in the abstract an informative and balanced summary of what was done and what was found	1-3
**Introduction**			
Background/rationale	2	Explain the scientific background and rationale for the investigation being reported	4
Objectives	3	State specific objectives, including any prespecified hypotheses	4
**Methods**			
Study design	4	Present key elements of study design early in the paper	5
Setting	5	Describe the setting, locations, and relevant dates, including periods of recruitment, exposure, follow-up, and data collection	5-6
Participants	6	(*a*) Give the eligibility criteria, and the sources and methods of selection of participants. Describe methods of follow-up<br>(*b*) For matched studies, give matching criteria and number of exposed and unexposed	5-6, SM
Variables	7	Clearly define all outcomes, exposures, predictors, potential confounders, and effect modifiers. Give diagnostic criteria, if applicable	5-8, SM
Data sources/ measurement	8*	For each variable of interest, give sources of data and details of methods of assessment (measurement). Describe comparability of assessment methods if there is more than one group	5-8, SM
Bias	9	Describe any efforts to address potential sources of bias	6-8, SM
Study size	10	Explain how the study size was arrived at	5-6
Quantitative variables	11	Explain how quantitative variables were handled in the analyses. If applicable, describe which groupings were chosen and why	SM
Statistical methods	12	(*a*) Describe all statistical methods, including those used to control for confounding<br>(*b*) Describe any methods used to examine subgroups and interactions<br>(*c*) Explain how missing data were addressed<br>(*d*) If applicable, explain how loss to follow-up was addressed<br>(*e*) Describe any sensitivity analyses	7-9, SM
**Results**			
Participants	13*	(a) Report numbers of individuals at each stage of study—eg numbers potentially eligible, examined for eligibility, confirmed eligible, included in the study, completing follow-up, and analysed<br>(b) Give reasons for non-participation at each stage<br>(c) Consider use of a flow diagram	9-11
Descriptive data	14*	(a) Give characteristics of study participants (eg demographic, clinical, social) and information on exposures and potential confounders<br>(b) Indicate number of participants with missing data for each variable of interest<br>(c) Summarise follow-up time (eg, average and total amount)	Table 1
Outcome data	15*	Report numbers of outcome events or summary measures over time	9-11

Main results	16	(*a*) Give unadjusted estimates and, if applicable, confounder-adjusted estimates and their precision (eg, 95% confidence interval). Make clear which confounders were adjusted for and why they were included<br>(*b*) Report category boundaries when continuous variables were categorized<br>(*c*) If relevant, consider translating estimates of relative risk into absolute risk for a meaningful time period	9-10, Fig 1
Other analyses	17	Report other analyses done—eg analyses of subgroups and interactions, and sensitivity analyses	10-11, Table 2, Fig 2, SM
**Discussion**			
Key results	18	Summarise key results with reference to study objectives	11-12
Limitations	19	Discuss limitations of the study, taking into account sources of potential bias or imprecision. Discuss both direction and magnitude of any potential bias	13-15
Interpretation	20	Give a cautious overall interpretation of results considering objectives, limitations, multiplicity of analyses, results from similar studies, and other relevant evidence	12-14
Generalisability	21	Discuss the generalisability (external validity) of the study results	14-15
**Other information**			
Funding	22	Give the source of funding and the role of the funders for the present study and, if applicable, for the original study on which the present article is based	16

*Give information separately for exposed and unexposed groups.

Note: An Explanation and Elaboration article discusses each checklist item and gives methodological background and published examples of transparent reporting. The STROBE checklist is best used in conjunction with this article (freely available on the Web sites of PLoS Medicine at http://www.plosmedicine.org/, Annals of Internal Medicine at http://www.annals.org/, and Epidemiology at http://www.epidem.com/). Information on the STROBE Initiative is available at http://www.strobe-statement.org.

Supplementary Tables

Table 1: Specification of the target trial and emulation of this using electronic health records

Protocol components	Target trial specification	Emulation of the target trial
Eligibility criteria - inclusion	Individuals over the age of 18	Individuals over the age of 18
Eligibility criteria - exclusion	Pre-existing diagnoses of common and / or serious mental illness, as defined as any of F20-F29 (schizophrenia, schizotypal, delusional, and other non-mood psychotic disorders), F30-F39 (mood [affective] disorders), F40-F48 (anxiety, dissociative, stress-related, somatoform and other nonpsychotic mental disorders), or F01-F09 (mental disorders due to known physiological conditions including cognitive disorders).	Pre-existing diagnoses of common and / or serious mental illness recorded in a patient's health record, as defined as any of F20-F29 (schizophrenia, schizotypal, delusional, and other non-mood psychotic disorders), F30-F39 (mood [affective] disorders), F40-F48 (anxiety, dissociative, stress-related, somatoform and other nonpsychotic mental disorders), or F01-F09 (mental disorders due to known physiological conditions including cognitive disorders).<br><br>We also ran robustness analyses with additional exclusions of contraindications for study medication, previous use of prucalopride in comparator cohorts, overlap with other study arm following index prescription, neuropsychiatric disease, and recent prescription of the two most frequently prescribed SSRI antidepressants.
Treatment assignment	Patients randomly assigned to prucalopride, linaclotide or lubiprostone	We assume random assignment of treatment as all three medications are third line approved medications for chronic constipation.<br><br>All cohorts were also matched using propensity score matching for disorders and medication that could be associated with differences in choices of anti-constipation treatment and / or with psychiatric disorders. In addition, matching occurred for any diagnoses or medication where the baseline incidence was greater than 5% in both cohorts and the difference between cohorts greater than 2% at the pre-matching stage.<br><br>Baseline characteristics of cohorts prior to matching for 121 variables (lifetime, 5 years and 1 year before inclusion) were checked.
Treatment	Treatment initiated at baseline (first prescription)<br><br>Prescription fulfilled at outpatient pharmacy with records accessible to study team	Treatment initiated at baseline (first prescription recorded in the patient's health record)<br><br>Prescription information identified from TriNetX US Collaborative Network.
Follow-up	12 months follow-up starts from date of first prescription (index date)	12 month follow-up starts from the date of first prescription (index date)
Outcomes	The incidence of a first diagnosis of major depressive disorder (F32) within one year of the index date	The incidence of a first diagnosis of major depressive disorder (F32) recorded in the patient's health record within one year of the index date
Causal contrasts of interest	Intention to treat analysis	Intention to treat analysis
Analysis plan to estimate causal contrasts of interest	Effectiveness of prucalopride on longitiduinal incidence of depression compared to comparators estimated as a cumulative incidence over the one-year outcome period using the Kaplan-Meier estimator. The Cox proportional hazard model and the log-rank test would be used to compare matched cohorts in terms of hazard ratios (HR) with 95% confidence intervals.	Effectiveness of prucalopride on future incidence of depression compared to comparators estimated as a cumulative incidence over the one-year outcome period using the Kaplan-Meier estimator. The Cox proportional hazard model and the log-rank test are used to compare matched cohorts in terms of hazard ratios (HR) with 95% confidence intervals.

Table 2: Baseline comparison statistics used for matching in primary analyses

TriNetX identifier / ICD-10 code	
AI	Age at Index
F	Female
2106-3	White
2186-5	Not Hispanic or Latino
UN	Unknown Ethnicity
M	Male
2131-1	Unknown Race
2054-5	Black or African American
UN	Unknown Gender
M	Married
S	Never married
D	Divorced
L	Legally separated
W	Widowed
T	Domestic partner
K59	Other functional intestinal disorders
K21	Gastro-esophageal reflux disease
I10	Essential (primary) hypertension
N80-N98	Noninflammatory disorders of female genital tract
K58	Irritable bowel syndrome
K31.84	Gastroparesis
E78	Disorders of lipoprotein metabolism and other lipidemias
H00-H59	Diseases of the eye and adnexa
D10-D36	Benign neoplasms, except benign neuroendocrine tumors
K63	Other diseases of intestine
E00-E07	Disorders of thyroid gland
G47	Sleep disorders
G89.2	Chronic pain, not elsewhere classified
E50-E64	Other nutritional deficiencies
E08-E13	Diabetes mellitus
J00-J06	Acute upper respiratory infections
M15-M19	Osteoarthritis
K40-K46	Hernia
E66	Overweight and obesity
G43	Migraine
M62	Other disorders of muscle
D64	Other anemias
I70-I79	Diseases of arteries, arterioles and capillaries
M30-M36	Systemic connective tissue disorders
I20-I25	Ischemic heart diseases
K80-K87	Disorders of gallbladder, biliary tract and pancreas

I49	Other cardiac arrhythmias
D50	Iron deficiency anemia
N17-N19	Acute kidney failure and chronic kidney disease
K90	Intestinal malabsorption
F17	Nicotine dependence
G90	Disorders of autonomic nervous system
Z79.891	Long term (current) use of opiate analgesic
G25	Other extrapyramidal and movement disorders
G35-G37	Demyelinating diseases of the central nervous system
C15-C26	Malignant neoplasms of digestive organs
G50-G59	Nerve, nerve root and plexus disorders
F10-F19	Mental and behavioural disorders due to psychoactive substance use
T50.901	Poisoning by unspecified drugs etc. (accidental)
T50.902	Poisoning by unspecified drugs etc. (intentional self-harm)
G20	Parkinson's disease
G30-G32	Other degenerative diseases of the nervous system
CN100	ANALGESICS
TN400	ELECTROLYTES/MINERALS
GA200	LAXATIVES
CN101	OPIOID ANALGESICS
CN103	NON-OPIOID ANALGESICS
HS050	ADRENAL CORTICOSTEROIDS
26225	ondansetron
CN300	SEDATIVES/HYPONTICS
AU000	AUTONOMIC MEDICATIONS
GA202	HYPEROSMOTIC LAXATIVES
RE100	ANTIASTHMA/BRONCHODILATORS
CN600	ANTIDEPRESSANTS
CV300	ANTIARRHYTHMICS
GA100	ANTACIDS
40790	pantoprazole
GA300	ANTIULCER AGENTS
CN400	ANTICONVULSANTS
GA204	STIMULANT LAXATIVES
CV350	ANTILIPEMIC AGENTS
7646	omeprazole
HS500	BLOOD GLUCOSE REGULATION AGENTS
4337	fentanyl
IM100	VACCINES
CV100	BETA BLOCKERS/RELATED
7804	oxycodone
CV700	DIURETICS
HS900	HORMONES/SYNTHETICS/MODIFIERS,OTHER
GA205	STOOL SOFTENER

AH100	ANTIHISTAMINES,PHENOTHIAZINE
AH105	ANTIHISTAMINES,PIPERAZINE
5489	hydrocodone
HS850	THYROID MODIFIERS
10689	tramadol
3423	hydromorphone
CV200	CALCIUM CHANNEL BLOCKERS
CN601	TRICYCLIC ANTIDEPRESSANTS
623033	lubiprostone
7052	morphine
CN102	OPIOID ANTAGONIST ANALGESICS
CN105	ANTIMIGRAINE AGENTS
1873752	plecanatide
CV490	ANTIHYPERTENSIVES,OTHER
HS300	ESTROGENS
9796	simethicone
CV805	ANGIOTENSIN II INHIBITOR
CN800	CNS STIMULANTS
HS200	CONTRACEPTIVES,SYSTEMIC
CV800	ACE INHIBITORS
CN700	ANTIPSYCHOTICS
72625	duloxetine
10737	trazodone
283742	esomeprazole
HS800	PROGESTINS
8704	prochlorperazine
2670	codeine
9601	scopolamine
GA208	ANTIDIARRHEAL AGENTS
CV250	ANTIANGINALS
42347	bupropion
816346	dexlansoprazole
36437	sertraline
321988	escitalopram
15996	mirtazapine
39786	venlafaxine
4493	fluoxetine
2556	citalopram
32937	paroxetine
3626	domperidone

Table 3: Baseline characteristics before matching for cohorts involved in primary analyses. Comorbidities and medications are lifetime prevalence

	**Prucalopride**	**Lubiprostone**	**Linaclotide**
Cohort size (n)	8694	80124	174902
Age at index (y, SD)	48.8, 19.0	54.6, 19.7	54.9, 18.1
Sex (M: F, unknown / other)	20.6, 75.8, 3.6	26.5, 70.2, 3.4	23.8, 72.7, 3.5
Ethnicity (W, B, unknown / other)	71.8, 9.5, 18.7	65.6, 15.1, 19.3	61.8, 19.3, 18.9
Marital status (married / partner, divorced / separated, widowed, never married, unknown)	20.4, 3.6, 2.5, 12.5, 61.0		29.7, 6.4, 6.7, 14.0, 43.2
COMORBIDITIES, %			
Gastro-oesophageal reflux disease	35.7	20.8	21.5
Primary hypertension	20.2	25.8	26.3
IBS	19.4	10.8	10.5
Lipidaemias	18.6	21.7	22.7
Disorders of thyroid gland	14.0	11.2	11.8
Diabetes mellitus	11.8	13.2	13.6
Chronic pain	13.4	9.5	10.1
Overweight, obesity	10.0	9.6	11.3
Ischaemic heart diseases	7.3	9.2	8.8
Iron deficiency anaemia	7.0	3.9	4.4
Acute and chronic kidney disease	5.6	7.0	6.9
Digestive malignancy	1.1	1.4	1.1
Demyelinating diseases of the nervous system	1.6	1.2	1.1
Parkinson's disease	0.9	1.2	1.0
MEDICATIONS, %			
Laxatives	54.5	52.7	47.3
Opioid analgesics	50.0	56.1	51.5
Sedatives / hypnotics	47.4	45.10	41.0
Corticosteroids	48.1	43.7	45.6
Antidepressants	40.8	38.2	35.4
Antilipaemic agents	26.5	34.5	34.8
Stimulant laxatives	30.9	28.1	24.3
Stool softeners	19.8	24.9	20.7
Thyroid modifiers	16.2	16.4	16.3
Antipsychotics	9.0	10.0	7.6
Duloxetine	8.9	8.0	7.5
Trazodone	8.2	9.8	8.3
Sertraline	4.8	4.7	4.7
Escitalopram	5.3	7.8	5.0
Mirtazapine	4.6	2.7	2.5
Fluoxetine	3.4	2.9	3.0
Citalopram	2.3	3.3	2.9

Table 4: Comorbidities and medications before matching for prucalopride versus linaclotide cohorts involved in primary analyses, restricted to 5 years and 1 year before analysis

	**5 years before**		**1 year before**	
COMORBIDITIES, %	**Prucalopride**	**Linaclotide**	**Prucalopride**	**Linaclotide**
Gastro-oesophageal reflux disease	33.6	19.5	26.4	15.3
Primary hypertension	18.6	24.3	13.6	19.8
IBS	18.5	10.3	14.5	8.7
Lipidaemias	16.8	20.3	12.2	16.6
Disorders of thyroid gland	12.6	10.5	8.6	8.1
Diabetes mellitus	11.1	12.5	9.3	10.6
Chronic pain	12.6	9.1	7.7	6.5
Overweight, obesity	9.2	8.9	5.7	7.3
Ischaemic heart diseases	6.5	8.6	4.3	6.2
Acute and chronic kidney disease	5.2	6.7	3.6	5.3
Digestive malignancy	1.0	1.3	0.7	0.8
Demyelinating diseases of the nervous system	1.4	1.1	1.0	0.8
Parkinson's disease	0.8	1.2	0.7	0.8
MEDICATIONS, %				
Laxatives	52.2	51.4	38.1	35.2
Opioid analgesics	46.0	54.2	32.2	38.0
Sedatives / hypnotics	43.8	43.2	29.7	28.7
Corticosteroids	45.9	42.1	32.7	33.9
Antidepressants	38.4	37.2	29.6	29.3
Antilipaemic agents	24.9	33.6	17.2	27.3
Stimulant laxatives	28.7	27.0	18.2	15.4
Stool softeners	16.9	23.4	10.6	12.9
Thyroid modifiers	15.4	16.1	10.9	13.5
Antipsychotics	8.4	9.7	6.2	5.3
Duloxetine	8.3	7.7	5.5	6.1
Trazodone	7.6	9.4	5.9	6.2
Sertraline	4.4	4.4	3.7	3.4
Escitalopram	5.0	4.6	3.3	3.7
Mirtazapine	4.4	2.6	3.9	2.1
Fluoxetine	3.0	2.7	2.5	2.3
Citalopram	1.9	3.0	1.6	2.2

Table 5: Comorbidities and medications before matching for prucalopride versus lubiprostone cohorts involved in primary analyses, restricted to 5 years and 1 year before analysis

	**5 years before**		**1 year before**	
COMORBIDITIES, %	**Prucalopride**	**Lubiprostone**	**Prucalopride**	**Lubiprostone**
Gastro-oesophageal reflux disease	33.6	19.5	26.4	15.0
Primary hypertension	18.6	24.3	13.6	19.8
IBS	18.5	10.3	14.5	9.0
Lipidaemias	16.8	20.3	12.2	15.8
Disorders of thyroid gland	12.6	10.5	8.6	8.0
Diabetes mellitus	11.1	12.5	9.3	10.4
Chronic pain	12.6	9.1	7.7	6.5
Overweight, obesity	9.2	8.9	5.7	6.3
Ischaemic heart diseases	6.5	8.6	4.3	6.6
Acute and chronic kidney disease	5.2	6.7	3.6	5.5
Digestive malignancy	1.0	1.3	0.7	1.1
Demyelinating diseases of the nervous system	1.4	1.1	1.0	0.9
Parkinson's disease	0.8	1.2	0.7	1.0
MEDICATIONS, %				
Laxatives	52.2	51.4	39.0	44.0
Opioid analgesics	46.0	54.2	30.6	44.1
Sedatives / hypnotics	43.8	43.2	29.6	34.2
Corticosteroids	45.9	42.1	31.6	33.0
Antidepressants	38.4	37.2	29.1	31.6
Antilipaemic agents	24.9	33.6	18.2	28.7
Stimulant laxatives	28.7	27.0	19.1	21.9
Stool softeners	16.9	23.4	9.9	18.1
Thyroid modifiers	15.4	16.1	11.4	14.0
Antipsychotics	8.4	9.7	5.8	8.0
Duloxetine	8.3	7.7	5.6	6.2
Trazodone	7.6	9.4	5.8	7.8
Sertraline	4.4	4.4	3.0	3.5
Escitalopram	5.0	4.6	3.3	3.6
Mirtazapine	4.4	2.6	3.4	2.2
Fluoxetine	3.0	2.7	2.1	2.1
Citalopram	2.0	3.0	1.4	2.3

Table 6: Results for the different robustness analyses in terms of incidence and hazard ratios of depression diagnosis.

Bold values for hazard ratios and p-values highlight statistically significant results.

	**Prucalopride versus linaclotide**					**Prucalopride versus lubiprostone**				
	**Prucalopride incidence, % (95% CI)**	**Linaclotide incidence, % (95% CI)**	**Hazard ratio (95% CI)**	**P value**	**Cohort size**	**Prucalopride incidence, % (95% CI)**	**Lubiprostone incidence, % (95% CI)**	**Hazard ratio (95% CI)**	**P value**	**Cohort size**
Excluding those with history of contraindications	5.57 (5.02-6.17)	6.98 (6.39-7.62)	**0.80 (0.69-0.91)**	**0.0012**	8119	5.56 (5.01-6.17)	6.88 (6.30-7.52)	**0.80 (0.70-0.92)**	**0.0021**	7908
Excluding those with prucalopride 1y prior to first prescription of comparator agent	5.64 (5.10-6.23)	6.53 (5.97-7.14)	**0.86 (0.75-0.99)**	**0.030**	8465	5.46 (4.92-6.05)	6.89 (6.31-7.52)	**0.79 (0.69-0.91)**	**<0.001**	8149
Excluding those with overlap of alternate drug in 1y from index date	5.71 (5.10-6.38)	6.66 (6.04-7.35)	**0.84 (0.72-0.98)**	**0.0029**	6836	5.38 (4.82-6.00)	6.58 (5.99-7.22)	**0.82 (0.71-0.95)**	**0.0073**	7501
Excluding those with previous neuropsychiatric illness	5.80 (5.24-6.41)	6.63 (6.05-7.26)	0.87 (0.76-1.00)	0.058	8177	5.71 (5.15-6.33)	6.72 (6.14-7.35)	**0.85 (0.74-0.97)**	**0.019**	7909
Excluding those recently prescribed one of the two most common SSRIs prior to index date	5.50 (4.96-6.09)	6.48 (5.92-7.09)	**0.85 (0.74-0.98)**	**0.023**	8413	5.45 (4.91-6.05)	6.43 (5.87-7.04)	**0.85 (0.74-0.98)**	**0.023**	8125

Table 7: Excluding those with contraindications for study medications prior to index date

Outcomes for prucalopride versus linaclotide / lubiprostone: percentage with each diagnosis during the 1-year outcome period and hazard ratio. Incidence shown with prior diagnosis of mental illness. Bold indicates significant difference between cohorts in HRs. NCO = composite negative control outcome

	**Prucalopride versus linaclotide**			**Prucalopride versus lubiprostone**		
	**Prucalopride incidence (%)**	**Linaclotide incidence (%)**	**Hazard ratio (95% CI)**	**Prucalopride incidence (%)**	**Lubiprostone incidence (%)**	**Hazard ratio (95% CI)**
Mood disorder	7.00 (6.39-7.67)	8.75 (8.09-9.46)	**0.80 (0.71-0.90)**	6.95 (6.34-7.63	8.62 (7.97-9.32)	**0.80 (0.71-0.91)**
Bipolar disorder	0.70 (0.52-0.94)	0.93 (0.73-1.19)	0.75 (0.51-1.10)	0.74 (0.55-0.99)	0.76(0.57-1.00)	0.99 (0.66-1.48)
Psychotic disorder	0.086 (0.039-0.19)	0.36 (0.24-0.54)	**0.25 (0.10-0.62)**	0.073 (0.03-0.18)	0.24 (0.15-0.40)	**0.33 (0.12-0.89)**
Dementia	0.36 (0.24-0.55)	0.46 (0.32-0.65)	0.75 (0.43-1.29)	0.37 (0.24-0.57)	0.39 (0.28-0.60)	0.93 (0.53-1.65)
Anxiety disorder	10.33 (9.60-11.12)	11.96 (11.20-12.77)	**0.87 (0.78-0.96)**	10.17 (9.43-10.97)	10.78(10.05-11.55)	0.95 (0.85-1.05)
Substance use disorder	2.64 (2.26-3.09)	3.28 (2.87-3.75)	0.79 (0.64-0.97)	2.56 (2.18-3.01)	2.89(2.50-3.33)	0.87 (0.70-1.08)
Any of NCOs	3.19 (2.75-3.71)	3.13 (2.71-3.61)	0.98 (0.80-1.21)	3.26 (2.80-3.79)	3.47 (3.03-3.97)	0.92 (0.75-1.13)

Table 8: Excluding those with a prescription of prucalopride prior to initiation of linaclotide or lubiprostone

Outcomes for prucalopride versus linaclotide / lubiprostone: percentage with each diagnosis during the 1-year outcome period and hazard ratio. Incidence shown with prior diagnosis of mental illness. Bold indicates significant difference between cohorts in HRs. NCO = composite negative control outcome

	**Prucalopride versus linaclotide**			**Prucalopride versus lubiprostone**		
	**Prucalopride incidence (%)**	**Linaclotide incidence (%)**	**Hazard ratio (95% CI)**	**Prucalopride incidence (%)**	**Lubiprostone incidence (%)**	**Hazard ratio (95% CI)**
Mood disorder	7.03 (6.43-7.68)	8.29 (7.67-8.97)	**0.85 (0.75-0.96)**	6.89 (6.29-7.55)	8.82 (8.17-9.52)	**0.78 (0.69-0.89)**
Bipolar disorder	0.66 (0.49-0.90)	0.77 (0.59-1.00)	0.86 (0.58-1.28)	0.73 (0.54-0.97)	0.88 (0.68-1.13)	0.84 (0.57-1.24)
Psychotic disorder	0.083 (0.037-0.19)	0.32 (0.21-0.48)	**0.28 (0.11-0.68)**	0.086 (0.039-0.19)	0.34 (0.23-0.51)	**0.26 (0.11-0.63)**
Dementia	0.35 (0.23-0.53)	0.53 (0.38-0.74)	0.65 (0.38-1.11)	0.35 (0.23-0.53)	0.32 (0.21-0.49)	1.06 (0.58-1.93)
Anxiety disorder	10.28 (9.56-11.05)	11.40 (10.67-12.17)	0.90 (0.82-1.00)	10.22 (9.49-11.00)	10.71 (10.00-11.47)	0.96 (0.87-1.07)
Substance use disorder	2.59 (2.22-3.02)	3.08 (2.69-3.53)	0.83 (0.68-1.02)	2.60 (2.22-3.04)	3.14 (2.74-3.59)	0.82 (0.67-1.01)
Any of NCOs	3.34 (2.89-3.85)	3.40 (2.99-3.91)	0.95 (0.78-1.16)	3.39 (2.93-3.93)	3.63 (3.19-4.13)	0.91 (0.74-1.10)

Table 9: Excluding those with recorded overlap of comparison medication for 1 year following index date (i.e. prucalopride for comparator cohorts; linaclotide or lubiprostone for prucalopride cohort)

Outcomes for prucalopride versus linaclotide / lubiprostone: percentage with each diagnosis during the 1-year outcome period and hazard ratio. Incidence shown with prior diagnosis of mental illness. Bold indicates significant difference between cohorts in HRs. NCO = composite negative control outcome

	**Prucalopride versus linaclotide**			**Prucalopride versus lubiprostone**		
	**Prucalopride incidence (%)**	**Linaclotide incidence (%)**	**Hazard ratio (95% CI)**	**Prucalopride incidence (%)**	**Lubiprostone incidence (%)**	**Hazard ratio (95% CI)**
Mood disorder	6.97 (6.30-7.70)	8.24 (7.54-8.99)	**0.83 (0.73-0.96)**	6.76 (6.14-7.45)	8.27 (7.62-8.98)	**0.82 (0.72-0.93)**
Bipolar disorder	0.60 (0.42-0.85)	0.72 (0.53-0.98)	0.83 (0.52-1.33)	0.98 (0.62-1.55)	0.60 (0.43-0.83)	0.60 (0.44-0.82)
Psychotic disorder	0.068 (0.025-0.18)	0.26 (0.15-0.43)	**0.27 (0.089-0.81)**	0.094 (0.042-0.21)	0.31 (0.20-0.48)	**0.31 (0.13-0.78)**
Dementia	0.22 (0.14-0.43)	0.54 (0.38-0.77)	**0.38 (0.19-0.76)**	0.34 (0.22-0.54)	0.39 (0.26-0.58)	0.84 (0.46-1.52)
Anxiety disorder	9.76 (8.97-10.61)	11.52 (10.71-12.39)	**0.84 (0.75-0.95)**	9.99 (9.24-10.81)	10.70 (9.97-11.49)	0.93 (0.84-1.04)
Substance use disorder	2.51 (2.10-2.99)	2.94 (2.52-3.43)	0.83 (0.65-1.05)	2.39 (2.01-2.84)	2.72 (2.33-3.16)	0.87 (0.69-1.09)
Any of NCOs	3.30 (2.80-3.88)	3.39 (2.92-3.94)	0.92 (0.74-1.15)	3.24 (2.77-3.79)	3.17 (2.74-3.67)	1.01 (0.81-1.25)

Table 10: Exclusion of additional neuropsychiatric illness

Outcomes for prucalopride versus (i) linaclotide and (ii) lubiprostone: percentage with each diagnosis during the 1-year outcome period and hazard ratio. Incidence shown with no prior neuropsychiatric illness. Bold indicates significant difference between cohorts in HRs. NCO = composite negative control outcome

	**Prucalopride versus linaclotide**			**Prucalopride versus lubiprostone**		
	**Prucalopride incidence (%)**	**Linaclotide incidence (%)**	**Hazard ratio (95% CI)**	**Prucalopride incidence (%)**	**Lubiprostone incidence (%)**	**Hazard ratio (95% CI)**
Mood disorder	7.13 (6.52-7.80)	8.49 (7.84-9.19)	**0.84 (0.74-0.95)**	7.02 (6.40-7.70)	8.31 (7.67-9.01)	**0.84 (0.74-0.96)**
Bipolar disorder	0.67 (0.50-0.91)	0.99 (0.78-1.26)	0.70 (0.47-1.03)	0.68 (0.50-0.92)	0.68 (0.51-0.92)	1.01 (0.66-1.54)
Psychotic disorder	0.07 (0.029-0.17)	0.26 (0.16-0.42)	**0.28 (0.10-0.75)**	0.072 (0.03-0.18)	0.31 (0.20-0.48)	**0.24 (0.091-0.64)**
Dementia	0.32 (0.21-0.50)	0.42 (0.29-0.62)	0.79 (0.44-1.41)	0.33 (0.21-0.52)	0.34 (0.22-0.51)	0.99 (0.54-1.82)
Anxiety disorder	10.26 (9.53-11.05)	11.74 (10.98-12.54)	**0.88 (0.80-0.98)**	10.15 (9.41-10.95)	10.33 (9.62-11.09)	0.99 (0.89-1.10)
Substance use disorder	2.64 (2.26-3.09)	2.94 (2.55-3.39)	0.89 (0.72-1.10)	2.67 (2.28-3.13)	3.35 (2.93-3.83)	**0.78 (0.64-0.96)**
Any of NCOs	3.29 (2.83-3.81)	3.31 (2.88-3.80)	0.97 (0.79-1.19)	3.25 (2.79-3.78)	3.80 (3.34-4.33)	0.83 (0.68-1.02)

Table 11: Exclusion of two most common SSRIs

Outcomes for prucalopride versus (i) linaclotide and (ii) lubiprostone: percentage with each diagnosis during the 1-year outcome period and hazard ratio. Incidence shown with exclusion of two most common SSRIs for 6 months prior to index date. Bold indicates significant difference between cohorts in HRs. NCO = composite negative control outcome

	**Prucalopride versus linaclotide**			**Prucalopride versus lubiprostone**		
	**Prucalopride incidence (%)**	**Linaclotide incidence (%)**	**Hazard ratio (95% CI)**	**Prucalopride incidence (%)**	**Lubiprostone incidence (%)**	**Hazard ratio (95% CI)**
Mood disorder	6.85 (6.26-7.50)	8.48 (7.84-9.16)	**0.80 (0.71-0.91)**	6.81 (6.21-7.47)	8.06 (7.44-8.74)	**0.85 (0.75-0.97)**
Bipolar disorder	0.64 (0.47-0.87)	1.12 (0.89-1.39)	**0.56 (0.39-0.82)**	0.63 (0.46-0.86)	0.68 (0.51-0.91)	0.95 (0.62-1.46)
Psychotic disorder	0.083 (0.037-0.19)	0.30 (0.19-0.46)	**0.29 (0.12-0.72)**	0.057 (0.021-0.15)	0.29 (0.18-0.45)	**0.22 (0.073-0.63)**
Dementia	0.32 (0.20-0.49)	0.44 (0.31-0.63)	0.70 (0.40-1.24)	0.32 (0.20-0.50)	0.45 (0.32-0.66)	0.69 (0.39-1.23)
Anxiety disorder	10.03 (9.32-10.80)	11.00 (10.28-11.77)	0.92 (0.83-1.02)	9.89 (9.17-10.67)	10.29 (9.59-11.04)	0.98 (0.88-1.09)
Substance use disorder	2.54 (2.17-2.97)	3.01 (2.62-3.46)	0.81 (0.66-1.00)	2.60 (2.22-3.05)	2.97 (2.59-3.42)	0.87 (0.70-1.07)
Any of NCOs	3.33 (2.89-3.85)	3.10 (2.69-3.57)	1.06 (0.86-1.30)	3.32 (2.86-3.84)	3.45 (3.01-3.94)	0.94 (0.77-1.15)

Table 12: Negative control outcomes

Prucalopride versus (i) linaclotide and versus (ii) lubiprostone for each negative control outcome over 12 months follow up

	**Prucalopride versus linaclotide**			**Prucalopride versus lubiprostone**		
	**Prucalopride incidence (%)**	**Linaclotide incidence (%)**	**Hazard ratio (95% CI)**	**Prucalopride incidence (%)**	**Lubiprostone incidence (%)**	**Hazard ratio (95% CI)**
Ganglion	0.20 (0.12-0.35)	0.26 (0.16-0.41)	0.76 (0.37-1.56)	0.19 (0.11-0.34)	0.21 (0.13-0.36)	0.94 (0.43-2.03)
Ingrowing nail	0.29 (0.20-0.49)	0.31 (0.20-0.48)	1.01 (0.54-1.90)	0.30 (0.21-0.50)	0.27 (0.18-0.45)	1.16 (0.61-2.21)
Epidermal cyst	0.29 (0.20-0.49)	0.36 (0.24-0.54)	0.80 (0.43-1.47)	0.25 (0.18-0.48)	0.35 (0.24-0.53)	0.68 (0.36-1.30)
Senile keratosis	0.47 (0.32-0.67)	0.40 (0.27-0.58)	1.13 (0.67-1.92)	0.46 (0.32-0.67)	0.42 (0.29-0.60)	1.10 (0.65-1.85)
Trigger finger	0.34 (0.22-0.52)	0.47 (0.33-0.66)	0.72 (0.41-1.25)	0.32 (0.21-0.51)	0.43 (0.30-0.62)	0.72 (0.41-1.29)
Onycholysis	0.061 (0.023-0.16)	0.059 (0.022-0.16)	1.02 (0.25-4.06)	0.063 (0.023-0.17)	0.09 (0.04-0.20)	0.67 (0.19-2.39)
Viral warts	0.075 (0.031-0.18)	0.10 (0.049-0.22)	0.75 (0.24-2.38)	0.059 (0.022-0.16)	0.06 (0.022-0.16)	1.07 (0.27-4.30)
Cutaneous abscess	0.21 (0.12-0.36)	0.28 (0.18-0.44)	0.72 (0.36-1.46)	0.20 (0.11-0.35)	0.15 (0.092-0.30)	1.34 (0.58-3.11)
Otitis externa	0.44 (0.30-0.64)	0.43 (0.31-0.64)	1.02 (0.60-1.71)	0.46 (0.31-0.66)	0.58 (0.42-0.80)	0.79 (0.48-1.28)
Hand fracture	0.00 (0.012-0.19)	0.013 (0.0019-0.095)	4e-09 (0.00-Inf)	0.00 (0.012-0.20)	0.028 (0.0071-0.11)	3.9e-09 (0.00-Inf)
Blepharitis	0.26 (0.16-0.43)	0.23 (0.18-0.45)	1.15 (0.57-2.32)	0.28 (0.17-0.45)	0.30 (0.20-0.47)	0.90 (0.47-1.73)
Adhesive capsulitis	0.24 (0.14-0.40)	0.18 (0.11-0.34)	1.23 (0.57-2.67)	0.23 (0.13-0.39)	0.22 (0.13-0.36)	0.94 (0.45-1.98)
Paronychia	0.31 (0.19-0.48)	0.16 (0.087-0.29)	1.85 (0.88-3.89)	0.30 (0.19-0.48)	0.24 (0.18-0.44)	1.28 (0.65-2.50)
Knee injury	0.039 (0.0096-0.15)	0.012 (0.0017-0.087)	2.70 (0.24-30.19)	0.04 (0.01-0.16)	0.03 (0.0075-0.12)	1.01 (0.14-7.26)
Insect bite	0.21 (0.12-0.36)	0.11 (0.054-0.22)	1.72 (0.71-4.16)	0.22 (0.13-0.38)	0.22 (0.13-0.37)	0.95 (0.45-1.99)
Lateral epicondylitis	0.049 (0.016-0.15)	0.029 (0.0072-0.12)	1.61 (0.27-9.67)	0.051 (0.016-0.16)	0.072 (0.04-0.20)	0.58 (0.14-2.45)
Finger laceration	0.035 (0.045-0.27)	0.049 (0.025-0.18)	0.62 (0.10-3.75)	0.037 (0.047-0.28)	0.06 (0.032-0.19)	0.49 (0.089-2.68)
Seborrhoeic dermatitis	0.48 (0.34-0.69)	0.28 (0.18-0.43)	1.68 (0.95-2.99)	0.48 (0.33-0.69)	0.55 (0.41-0.78)	0.89 (0.55-1.45)
Nasal polyp	0.083 (0.046-0.23)	0.043 (0.033-0.19)	1.77 (0.42-7.43)	0.068 (0.037-0.22)	0.058 (0.021-0.15)	1.12 (0.28-4.46)
Folliculitis	0.26 (0.16-0.43)	0.25 (0.15-0.40)	0.99 (0.50-1.96)	0.27 (0.17-0.45)	0.33 (0.22-0.51)	0.80 (0.42-1.52)
Any of NCOs	3.39 (2.94-3.91)	3.14 (2.73-3.61)	1.05 (0.86-1.28)	3.38 (2.92-3.91)	3.80 (3.35-4.32)	0.88 (0.72-1.07)

Table 13: Lubiprostone versus linaclotide

Outcomes for lubiprostone versus linaclotide: percentage with each diagnosis during the 1-year outcome period and hazard ratio. Incidence shown without previous mental illness. Bold indicates significant difference between cohorts in HRs

	**Lubiprostone incidence (%)**	**Linaclotide incidence (%)**	**Hazard ratio (95% CI)**
Mood disorder	7.89 (7.69-8.10)	8.06 (7.85-8.27)	0.98 (0.94-1.02)
Depression	6.38 (6.20-6.57)	6.43 (6.24-6.62)	1.00 (0.95-1.04)
Bipolar disorder	0.74 (0.68-0.81)	0.83 (0.76-0.90)	0.89 (0.78-1.00)
Psychotic disorder	0.41 (0.37-0.46)	0.38 (0.33-0.43)	1.09 (0.92-1.30)
Dementia	0.85 (0.78-0.92)	0.93 (0.86-1.01)	0.92 (0.82-1.03)
Anxiety disorder	9.07 (8.85-9.29)	10.12 (9.88-10.35)	**0.89 (0.86-0.92)**
Substance use disorder	3.39 (3.25-3.53)	3.39 (3.25-3.54)	1.00 (0.94-1.06)
Any of NCOs	3.05 (2.92-3.20)	3.01 (2.87-3.15)	1.01 (0.95-1.08)

Table 14: Plecanatide versus linaclotide

Outcomes for plecanatide versus linaclotide: percentage with each diagnosis during the 1-year outcome period and hazard ratio. Incidence shown without previous mental illness. Bold indicates significant difference between cohorts in HRs

	**Plecanatide incidence (%)**	**Linaclotide incidence (%)**	**Hazard ratio (95% CI)**
Mood disorder	7.80 (6.67-9.11)	7.04 (6.00-8.26)	1.06 (0.84-1.34)
Depression	6.48 (5.45-7.70)	5.64 (4.71-6.75)	1.10 (0.85-1.42)
Bipolar disorder	0.45 (0.23-0.87)	0.74 (0.45-1.23)	0.60 (0.26-1.37)
Psychotic disorder	0.37 (0.18-0.78)	0.25 (0.10-0.61)	1.44 (0.46-4.54)
Dementia	0.47 (0.28-0.99)	0.72 (0.42-1.21)	0.64 (0.27-1.47)
Anxiety disorder	11.43 (10.08-12.95)	11.25 (9.93-12.72)	1.00 (0.83-1.20)
Substance use disorder	2.86 (2.18-3.75)	2.79 (2.15-3.62)	0.95 (0.65-1.39)
Any of NCOs	3.61 (2.80-4.64)	2.94 (2.24-3.86)	1.22 (0.84-1.77)

Table 15: Interrupted time series analysis

Trend in depression diagnosis or any negative control outcomes (NCOs) incidence (and absolute counts) in the 12 months after versus before the first prescription of prucalopride. Incidences are reported as number of cases divided by the total number of people who had at least one health encounter within each month. Bold indicates significant difference. Negative slope value indicates the outcome was less likely after versus before prucalopride prescription; positive slope value indicates the outcome was more likely after versus before prucalopride prescription.

	**Slope (95% CI)**	**Slope, p-value**	**Intercept (95% CI)**	**Intercept, p-value**
Depression (incidence), /10,000 people per month	-3.05 (-4.95, -1.14)	**0.0058**	-8.88 (-22.67, 4.91)	0.22
Any NCOs (incidence), /10,000 people per month	1.53 (0.12, 2.93)	**0.047**	-1.49 (-11.65, 8.68)	0.78
Depression (count), per month	-0.13 (-0.16, -0.093)	**7.1e-07**	-0.017 (-0.24, 0.20)	0.88
Any NCOs (count), per month	-0.028 (-0.066, 0.0098)	0.16	0.04 (-0.23, 0.31)	0.77
Depression (incidence), including all months	-5.74 (-8.58, -2.90)	**0.00077**	-18.52 (-38.19, 1.14)	0.08
Any NCOs (incidence), including all months	1.36 (0.16, 2.56)	**0.038**	-3.89 (-12.20, 4.43)	0.37

Table 16: Comparison of the prevalence of history of psychiatric disorders (ICD-10 F01-F09, F20-F29, F30-F39, or F40-F48) between those prescribed prucalopride and those prescribed a comparator drug.

Correct on 1st February 2024

	**No history of psychiatric disorder**	**History of psychiatric disorder**	**Odds:**<br><br>**N. with history/N. without history of psychiatric disorder**	**Odds ratio**<br><br>**Prucalopride vs. comparator**
Prucalopride	8694	9013	1.03	-
Linaclotide	175468	120925	0.69	1.49
Lubiprostone	80121	53484	0.67	1.53

Supplementary Figures

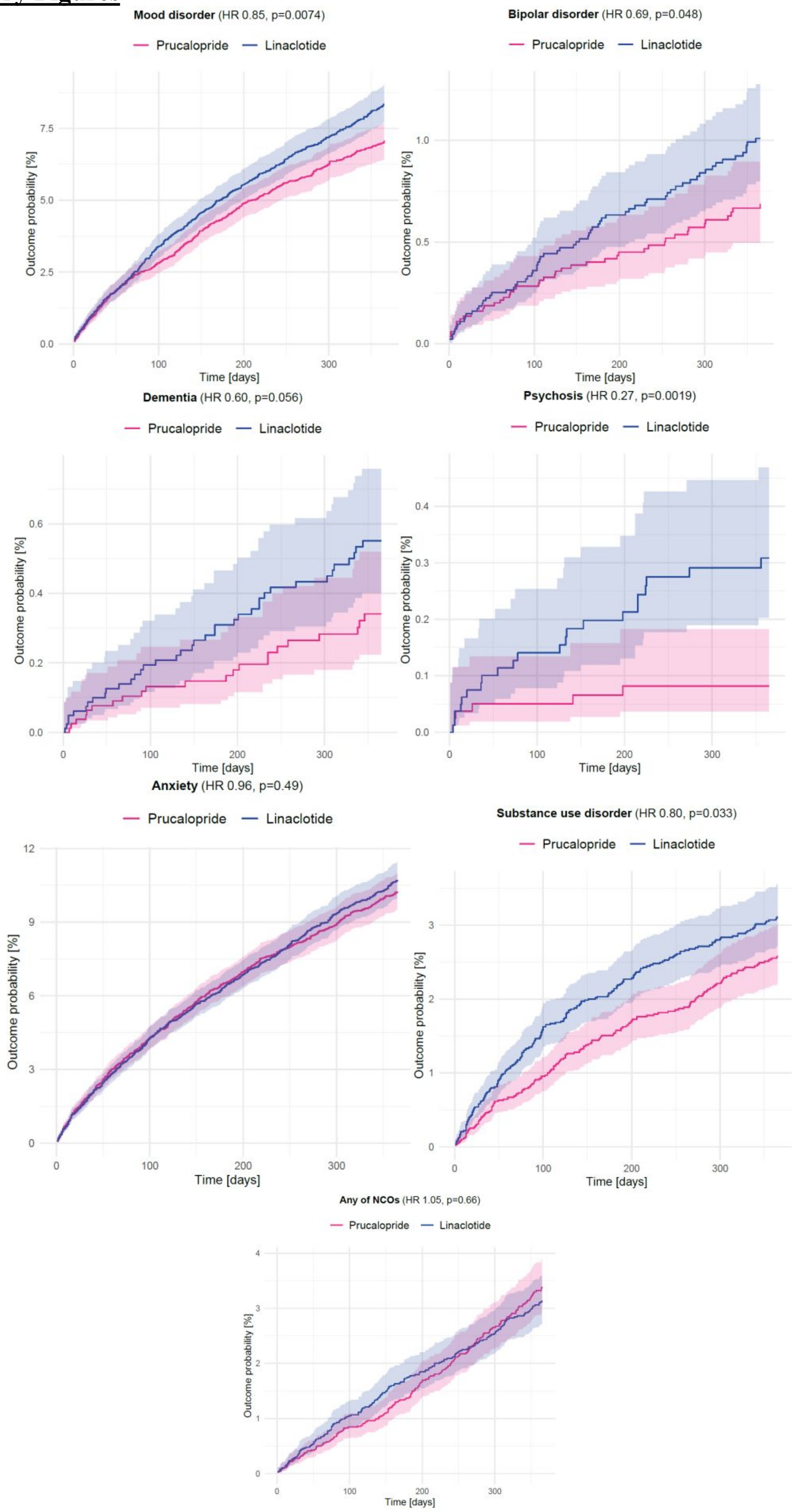

Figure 1: Kaplan-Meier curves showing outcome probability over 12 months for prucalopride versus linaclotide for each secondary mental health outcome and composite negative control outcome

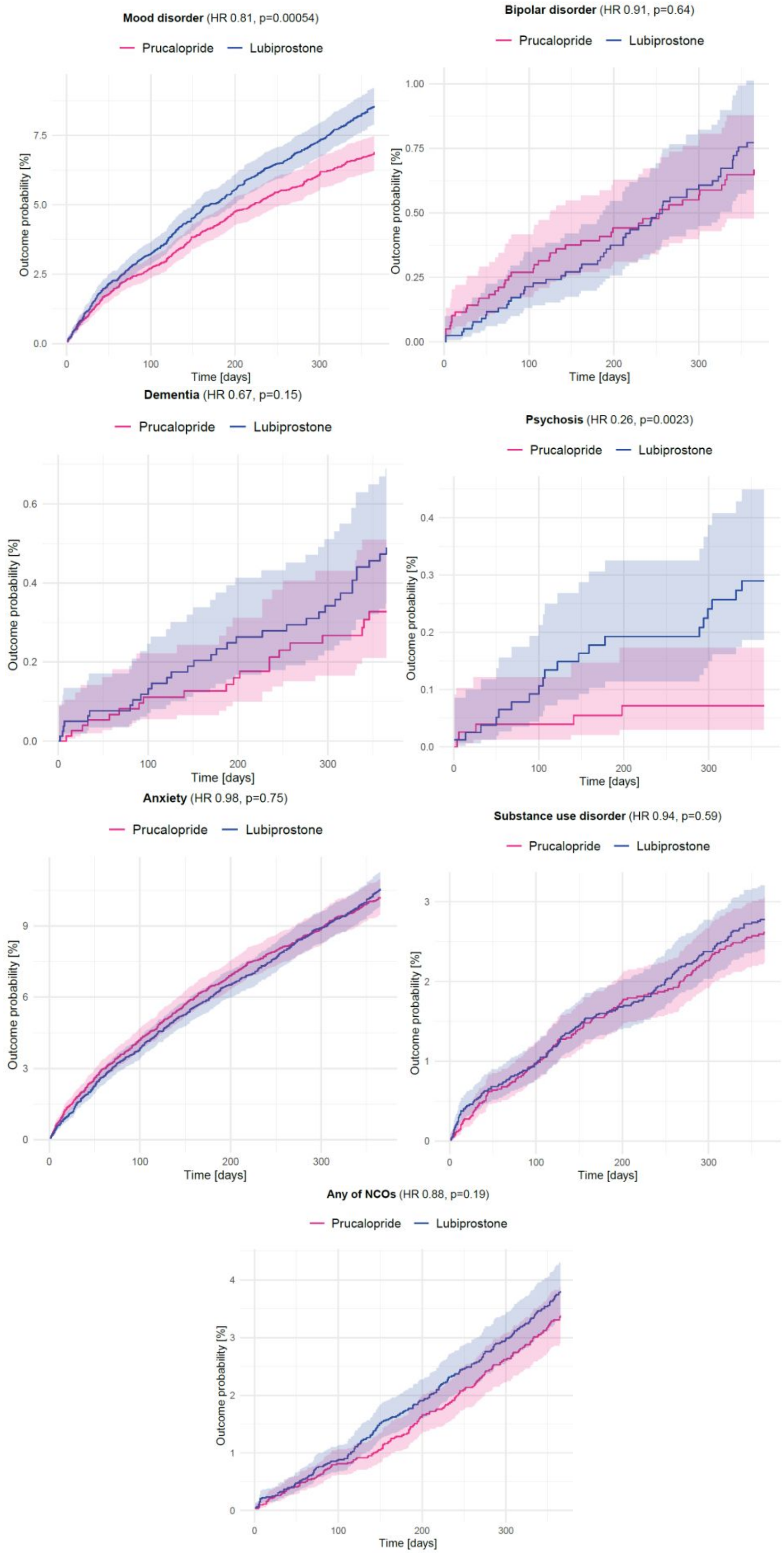

Figure 2: Kaplan-Meier curves showing outcome probability over 12 months for prucalopride versus lubiprostone for each secondary mental health outcome and composite negative control outcome

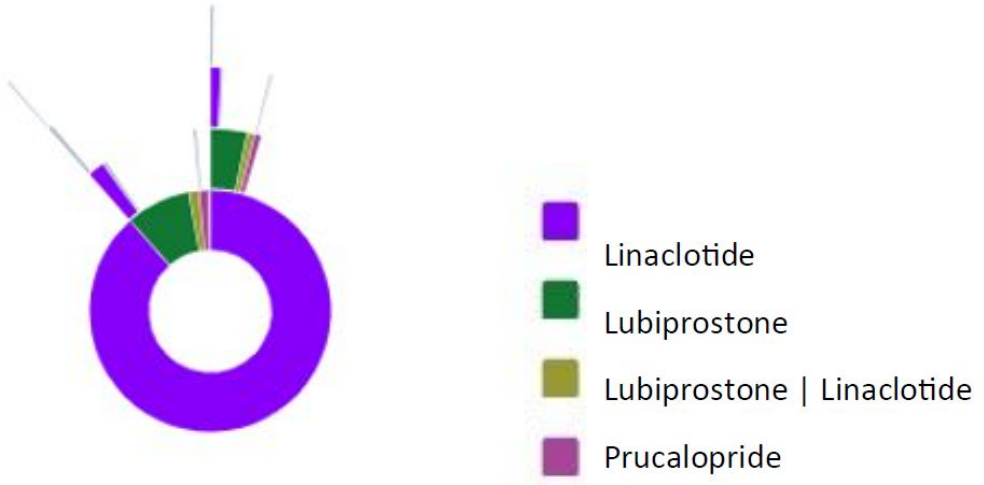

**Treatment 1**	**Treatment 2**	**% patients with this pathway**
linaclotide		84
linaclotide	lubiprostone	2.3
linaclotide	lubiprostone | linaclotide	0.5
linaclotide	prucalopride	0.4
linaclotide	other treatments	<0.1
lubiprostone		6.8
lubiprostone	linaclotide	1.2
lubiprostone	other treatments	0.3
lubiprostone | linaclotide		1.4
lubiprostone | linaclotide	other treatments	<0.1
prucalopride		0.99
prucalopride	other treatments	0.2
other treatments		0.2

(i) Following first prescription of linaclotide

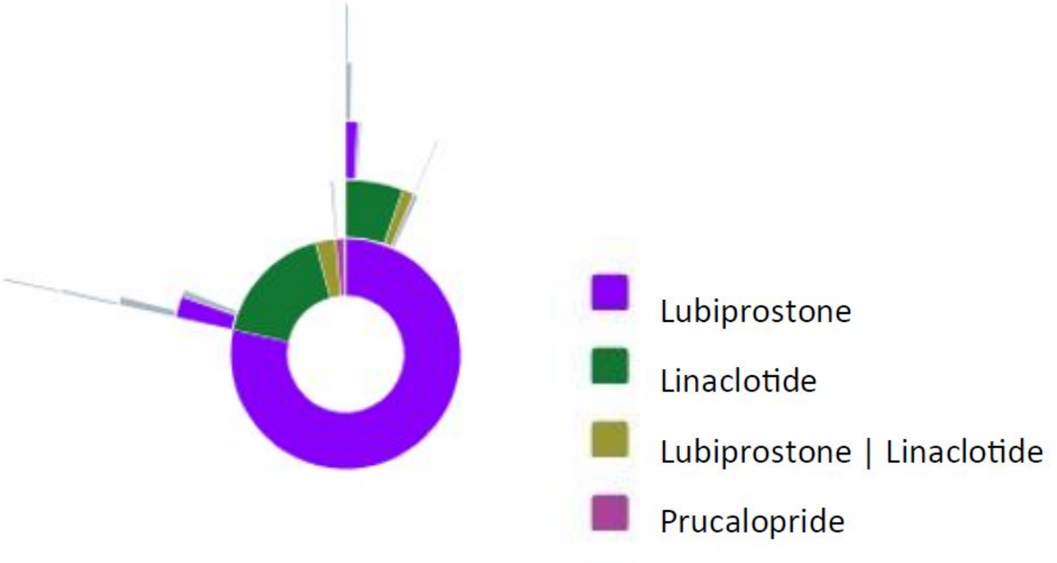

Treatment 1	Treatment 2	% of Patients with Pathway
linaclotide		15
linaclotide	lubiprostone	1.4
linaclotide	other treatments	0.6
lubiprostone		71
lubiprostone	linaclotide	4.3
lubiprostone	lubiprostone | linaclotide	1.1
lubiprostone	other treatments	0.3
lubiprostone | linaclotide		2.8
lubiprostone | linaclotide	other treatments	0.01
prucalopride		0.9
prucalopride	other treatments	0.2
other treatments		0.2

(ii) Following first prescription of lubiprostone

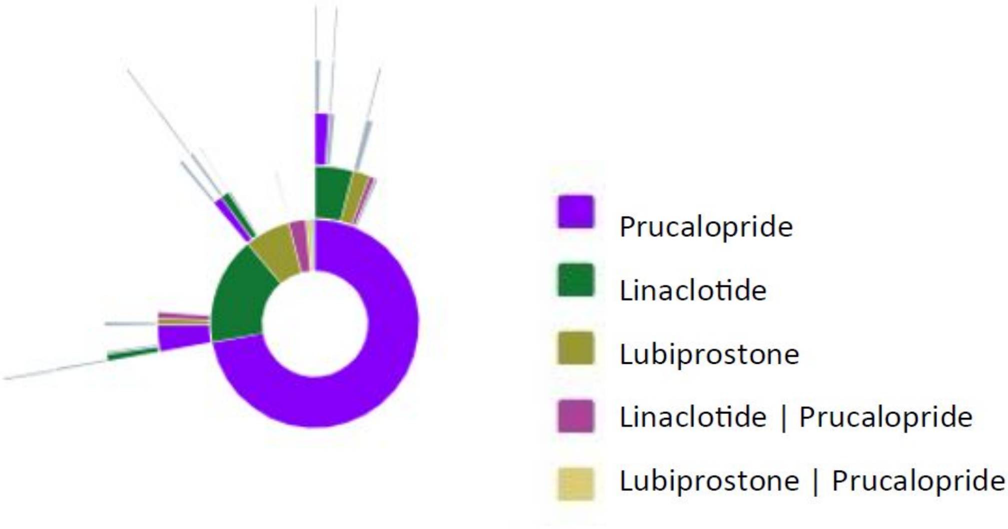

Treatment 1	Treatment 2	% of Patients with Pathway (2 s.f.)
linaclotide		13
linaclotide	linaclotide | prucalopride	0.6
linaclotide	lubiprostone	0.4
linaclotide	prucalopride	1.9
linaclotide	other treatments	0.5
linaclotide | prucalopride		2.5
linaclotide | prucalopride	other treatments	0.9
lubiprostone		4.9
lubiprostone	linaclotide	0.6
lubiprostone	prucalopride	0.7
lubiprostone	other treatments	0.2
lubiprostone | prucalopride		0.7
prucalopride		66
prucalopride	linaclotide	2.4
prucalopride	linaclotide | prucalopride	0.5
prucalopride	lubiprostone	1.2
prucalopride	other treatments	0.4
other treatments		0.6

(iii) Following first prescription of prucalopride

Figure 3: Sunburst plots showing participants in primary analysis cohorts who had subsequent prescriptions of the same and / or comparator agents over the following 1 year

For individuals within each cohort, the subsequent prescriptions that were recorded are shown. The inner ring shows the prescription immediately after the index; the outer "bursts" show third and subsequent prescriptions.

Secondary analyses: prucalopride vs (i) linaclotide (anxiety and bipolar disorder); (ii) lubiprostone (substance use disorder and any of NCOs)

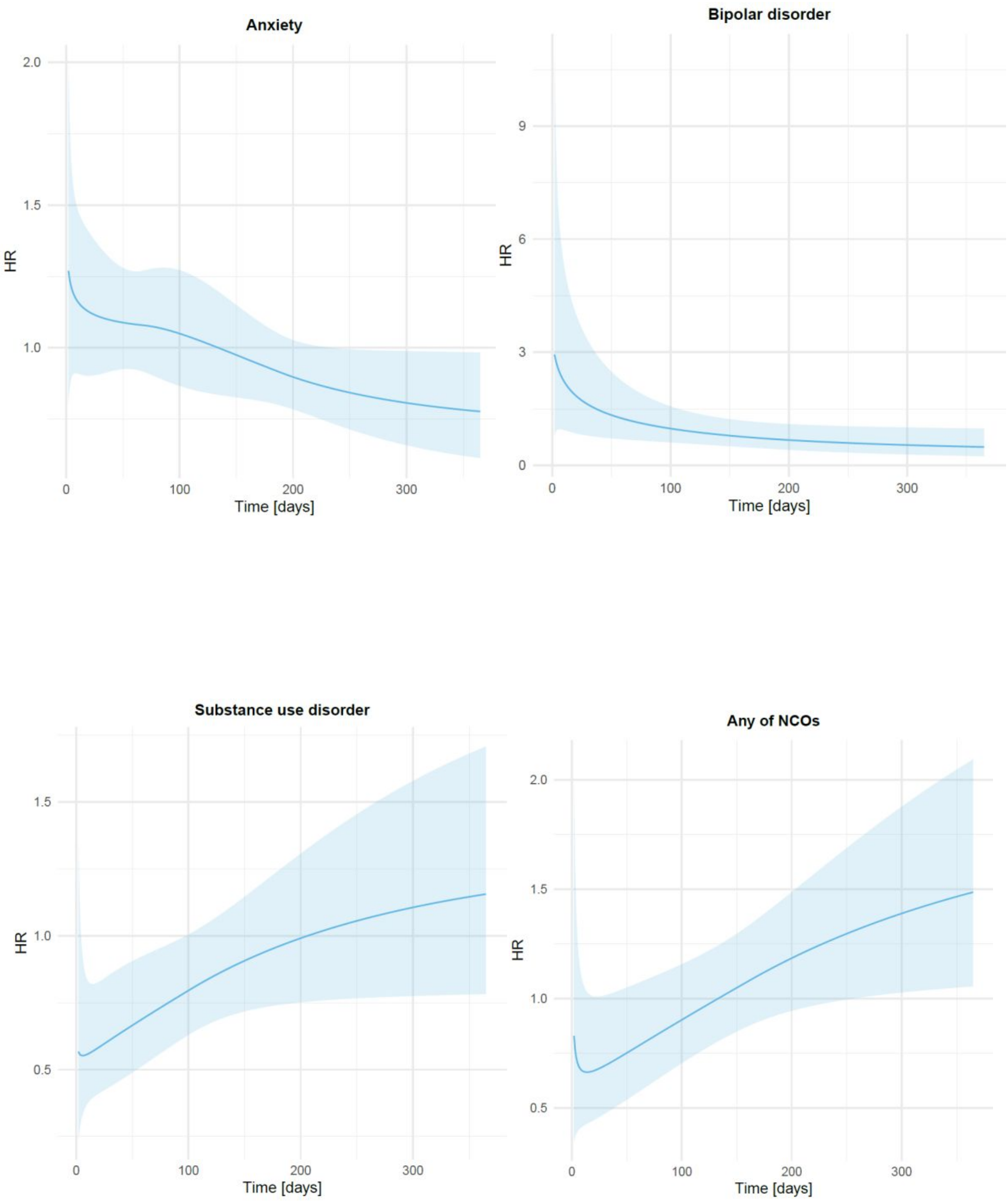

Figure 4: Mental health outcome time-varying hazard ratio where there was evidence of non-proportionality of hazards in the main comparison between the cohort of patients receiving prucalopride and a matched cohort receiving an alternative anti-constipation agent.

Shaded area represents a 95% confidence interval

Supplementary References

1. Taquet M, Dercon Q, Luciano S, Geddes JR, Husain M, Harrison PJ. Incidence, co-occurrence, and evolution of long-COVID features: A 6-month retrospective cohort study of 273,618 survivors of COVID-19. PLoS Med. 2021; 18(9): e1003773.
2. Taquet M, Luciano S, Geddes JR, Harrison PJ. Bidirectional associations between COVID-19 and psychiatric disorder: retrospective cohort studies of 62 354 COVID-19 cases in the USA. Lancet Psychiatry. 2021; 8(2): 130-40.
3. Hernán MA, Wang W, Leaf DE. Target Trial Emulation: A Framework for Causal Inference From Observational Data. JAMA. 2022.
4. Danaei G, Tavakkoli M, Hernán MA. Bias in observational studies of prevalent users: lessons for comparative effectiveness research from a meta-analysis of statins. 2012; 175(4): 250-62.
5. ASG. American Society of Gastroenterology: Pharmacological management of irritable bowel syndrome with constipation (IBS-C). 2022. https://gastro.org/clinical-guidance/pharmacological-management-of-irritable-bowel-syndrome-with-constipation-ibs-c/.
6. BSG. British Society of Gastroenterology Guidelines on the Management of Irritable Bowel Syndrome. 2021. https://www.bsg.org.uk/clinical-resource/british-society-of-gastroenterology-guidelines-on-the-management-of-irritable-bowel-syndrome/.
7. Burns EM, Rigby E, Mamidanna R, Bottle A, Aylin P, Ziprin P, et al. Systematic review of discharge coding accuracy. J Public Health (Oxf). 2012; 34(1): 138-48.
8. Nellesen D, Chawla A, Oh DL, Weissman T, Lavins BJ, Murray CW. Comorbidities in patients with irritable bowel syndrome with constipation or chronic idiopathic constipation: a review of the literature from the past decade. Postgrad Med. 2013; 125(2): 40-50.
9. Doshi JA, Cai Q, Buono JL, Spalding WM, Sarocco P, Tan H, et al. Economic burden of irritable bowel syndrome with constipation: a retrospective analysis of health care costs in a commercially insured population. J Manag Care Spec Pharm. 2014; 20(4): 382-90.
10. Haukoos JS, Lewis RJ. The Propensity Score. JAMA. 2015; 314(15): 1637-8.
11. Ali MS, Prieto-Alhambra D, Lopes LC, Ramos D, Bispo N, Ichihara MY, et al. Propensity Score Methods in Health Technology Assessment: Principles, Extended Applications, and Recent Advances. Frontiers in Pharmacology. 2019; 10.
12. Colbourne L, Harrison PJ. Brain-penetrant calcium channel blockers are associated with a reduced incidence of neuropsychiatric disorders. Mol Psychiatry. 2022.
13. Bernal JL, Cummins S, Gasparrini A. Interrupted time series regression for the evaluation of public health interventions: a tutorial. Int J Epidemiol. 2017; 46(1): 348-55.
14. VanderWeele TJ, Ding P. Sensitivity Analysis in Observational Research: Introducing the E-Value. Ann Intern Med. 2017; 167(4): 268-74.
